# Modular design workflow for 3D printable bioresorbable patient-specific bone scaffolds: extended features and clinical validation

**DOI:** 10.3389/fbioe.2024.1404481

**Published:** 2024-11-19

**Authors:** Buddhi Herath, Markus Laubach, Sinduja Suresh, Beat Schmutz, J. Paige Little, Prasad K. D. V. Yarlagadda, Heide Delbrück, Frank Hildebrand, Dietmar W. Hutmacher, Marie-Luise Wille

**Affiliations:** ^1^ Australian Research Council Training Centre for Multiscale 3D Imaging, Modelling, and Manufacturing, Queensland University of Technology, Brisbane, Australia; ^2^ Centre for Biomedical Technologies, Queensland University of Technology, Brisbane, Australia; ^3^ School of Mechanical, Medical and Process Engineering, Faculty of Engineering, Queensland University of Technology, Brisbane, Australia; ^4^ Jamieson Trauma Institute, Metro North Hospital and Health Service, Brisbane, Australia; ^5^ Department of Orthopaedics and Trauma Surgery, Musculoskeletal University Center Munich (MUM), LMU University Hospital, LMU Munich, Munich, Germany; ^6^ Biomechanics and Spine Research Group at the Centre for Children’s Health Research, Queensland University of Technology, Brisbane, Australia; ^7^ Department of Orthopaedics, Trauma and Reconstructive Surgery, RWTH Aachen University Hospital, Aachen, Germany; ^8^ Max Planck Queensland Centre for the Materials Science of Extracellular Matrices, Queensland University of Technology, Brisbane, Australia

**Keywords:** scaffold-guided bone regeneration, scaffold design workflow, additive manufacturing, generative design, parametric design

## Abstract

A previously in-house developed patient-specific scaffold design workflow was extended with new features to overcome several limitations and to broaden its adaptability to diverse bone defects, thereby enhancing its fit for routine clinical use. It was applied to three clinical cases for further validation. A virtual surgical resection tool was developed to remove regions of the bone defect models. The minor cavity fill module enabled the generation of scaffold designs with smooth external surfaces and the segmental defect fill module allowed a versatile method to fill a segmental defect cavity. The boundary representation method based surgical approach module in the original workflow was redeveloped to use functional representation, eliminating previously seen resolution dependant artefacts. Lastly, a method to overlay the scaffold designs on computed tomography images of the defect for design verification by the surgeon was introduced. The extended workflow was applied to two ongoing clinical case studies of a complex bilateral femoral defect and a humerus defect, and also to a case of a large volume craniomaxillofacial defect. It was able to successfully generate scaffolds without any obstructions to their surgical insertion which was verified by digital examination as well as using physical 3D printed models. All produced surface meshes were free from 3D printing mesh errors. The scaffolds designed for the ongoing cases were 3D printed and successfully surgically implanted, providing confidence in the extended modular workflow’s ability to be applied to a broad range of diverse clinical cases.

## 1 Introduction

Recently, scaffold-guided bone regeneration (SGBR) has been translated from *bench to bedside* for large bone defect reconstructions Laubach et al. (2022); Kobbe et al. (2020); Castrisos et al. (2022); Reichert et al. (2012); Cipitria et al. (2013). SGBR involves the surgical implantation of a three-dimensional (3D) printed biodegradable porous patient-specific implant (called a *“scaffold”*) loaded with autologous bone graft, leading to bone regeneration Zhou et al. (2023). Given the highly customised nature of patient-specific scaffolds, additive manufacturing (AM) has been identified to be a feasible manufacturing regime. Once a patient is presented to the clinic, the defect region is imaged using computed tomography (CT) and a digital 3D model of the bone defect is generated using image segmentation Jia et al. (2023). Through continuous consultation with the team of surgeons and the design engineer/manufacturer, a patient-specific scaffold design is created, 3D printed by certified manufacturers with a medical-grade bioresorbable polymer, sterilised and is finally surgically implanted Laubach et al. (2023).

A time efficient bottom-up design workflow has been recognised as a key facilitator for translating SGBR into a routine clinical practice Charbonnier et al. (2021). Towards this vision, a semi-automatic workflow was recently developed by our research group to design patient-specific SGBR scaffolds that are 3D printable^1^ Herath et al. (2023). It was developed within Rhinoceros 3D and Grasshopper (RhGh) software (Robert McNeel and Associates, Washington, United States) using their graphical programming environment employing its vast array of code components, organised into modules and clusters which form a cohesive design software. The primary input to the design workflow is the defect model computed via image segmentation of the patient’s CT images and the primary output is the exported surface mesh of the scaffolds design models along with their porosity and pore size distribution metrics.

To overcome the limitation of boundary representation (B-rep) modelling with respect to geometric Boolean operations producing mesh errors that hinders 3D printing Minetto et al. (2017); Kambampati et al. (2021), a dedicated Grasshopper plugin was developed based on OpenVDB Museth (2013), an opensource library which granted functional representation (F-rep) capabilities Kambampati et al. (2021); Ghadai et al. (2021). The plugin provides the seamless conversion between narrow-band level sets (F-rep representation of geometries) and polygonal surface meshes, and within the level set domain provide many geometric modelling functions such as Boolean operations, smoothing and offset functions among others. As a result, the output surface meshes of the designs are free from 3D printing mesh errors, namely, free from degenerate or self-intersecting faces and is watertight and manifold, thus being readily 3D printable without requiring manual post processing.

Modules were developed to create scaffolds designs which are patient-specific and can be inserted from a predetermined surgical approach without any obstructions from existing bone, semi-automatically generate surgical fixation points on scaffolds to securely fasten them to the host bone to prevent internal dislodgement and create scaffold designs based on the popular Voronoi tessellation Herath et al. (2021); Sharma et al. (2021); Chen et al. (2021); Klatt et al. (2019); Deering et al. (2021); Chen et al. (2019); Du et al. (2020), periodic lattice architectures Chen et al. (2020); Gatto et al. (2021); Onal et al. (2018) and triply periodic minimal surface (TPMS) Karakoç (2021); Shi et al. (2018); Günther et al. (2022); Poltue et al. (2021); Verma et al. (2022) based designs. A set of evaluation modules allow the calculation of porosity, distribution of pore sizes, virtual patient-specific fit, and check for surface mesh errors which prevent 3D printing. The plug-and-play nature of the workflow’s modules allows the user to quickly adapt it to different clinical cases. Finally, the workflow was validated with a clinical case study of a complex post-traumatic femoral shaft defect Herath et al. (2023).

The aim of the present research work is to extend the workflow by introducing new functionality to make it suitable for routine clinical use and to validate it accordingly in a series of clinical cases. The novelty of this work is that it adds important features which the previous workflow lacked but which were identified being crucial for clinical translation. The previous workflow did not have the built-in ability to resect portions of the bone defect model, create cavity fills for segmental defects by bridging the two open ends of the defect, avoid resolution dependant artefacts of the B-rep based Surgical Approach module, avoid sharp protrusions of the scaffold geometry which are likely to break during surgery, and a method to validate the scaffold design against the CT images of the patient. This study is about the development of these new features and their application to new clinical cases.

### 1.1 Extended SGBR scaffold design workflow

**FIGURE 1:**
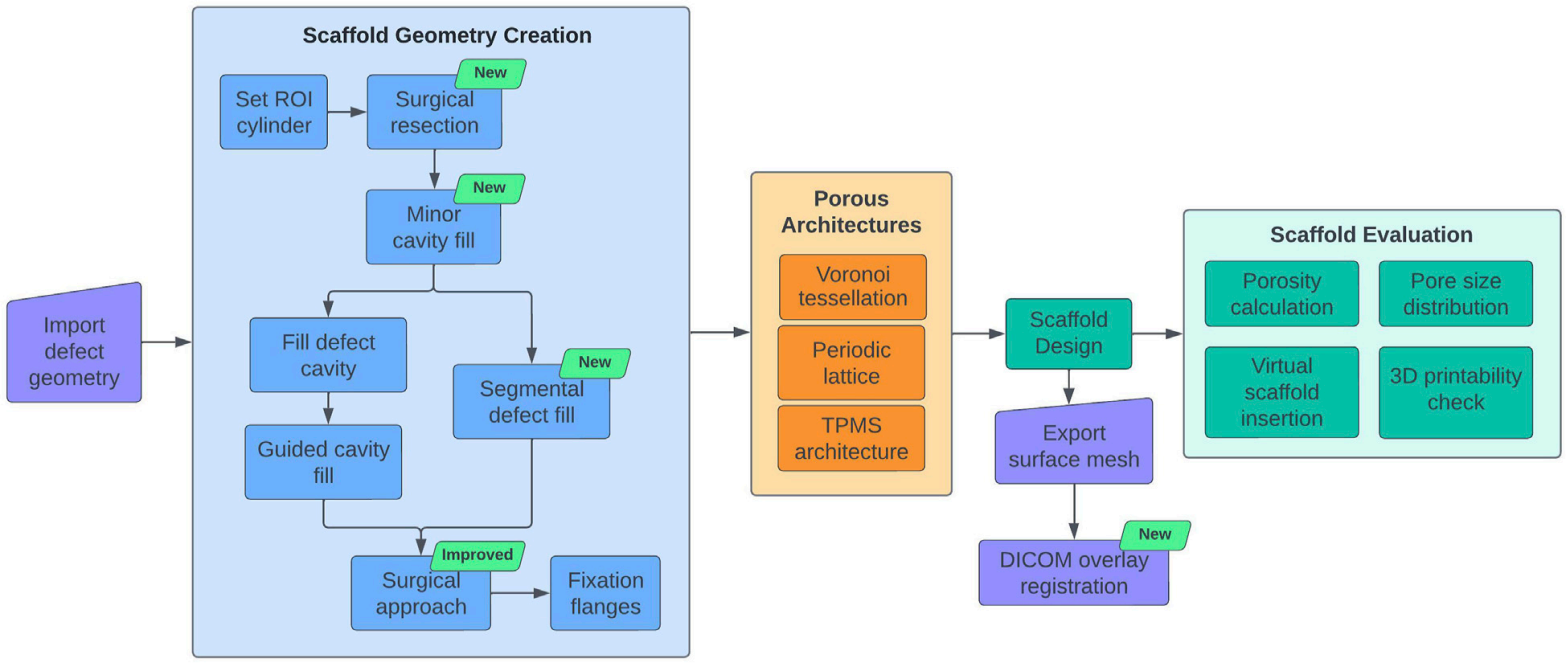
SGBR scaffold design workflow diagram. Newly developed modules are indicated by the green text box 'new'.

The workflow previously developed by this research group Herath et al. (2023) was extended with new modules and improvements to accommodate newly identified workflow requirements during routine clinical translation and to widen its capabilities. Figure 1 provides an overview of the original modules Herath et al. (2023) and the new modules developed in the course of this research. During surgery, it is frequently required to remove both malformed and/or sclerotic/necrotic bone as well as certain healthy bone areas in order to improve the regenerative capacity of the bone or the implantability of scaffolds. Ideally, these regions would be marked on the 3D defect model during the design stage. Therefore, it would be beneficial if the workflow had the ability to virtually resect regions of the bone defect model in real-time during the ongoing discussions with the surgeon. Hence, a Surgical Resection module was developed which allows the quick virtual resection of bone, screws or plate in the imported bone defect geometry.

Furthermore, the original Defect Cavity Fill module includes the creation of cavity fills using guide curves to cover portions of malformed bone. For non-complex segmental defects, however, we identified that the alternative method of interpolating between two intact cross-sections on either side of the defect provided superior cavity filling capabilities. Therefore, the Segmental Defect Fill module was developed to create patient-specific filling of a segmental bone defect by bridging any two cross-sections of bone selected by the user. Further, the Surgical Approach module is responsible for creating patient-specific implant geometries that can be surgically inserted from a predetermined direction of surgical entry (surgical approach) without any obstructions from existing bone. The Surgical Approach module was redeveloped to use F-rep modelling to avoid any artefacts generated by B-rep modelling, which improved its accuracy significantly.

The workflow creates geometrically highly accurate patient-specific implants. Although seen as advantageous, in the event of narrow crevices being present in the bone defect model which are often seen in trauma induced defects, the patient-specific implant would be generated with sharp protrusions to fill these provided they are in-line with the surgical approach (see Section 2.1.4 for an example). Although surgeons can insert them without host bone-derived obstruction as they were designed by the workflow, they prefer a smooth external surface without sharp protrusions to avoid interposition with soft tissue during implantation. The bone defect model is the primary input to the workflow which is the result of image segmentation that separates bone from soft tissue in the CT images. Hence, the scaffold generated for this bone defect model would assume the soft tissue to be empty regions, thus creating geometries to fill these in. This soft tissue (muscles, vasculature and nerve bundles) could still pose disturbances to narrow protrusions during insertion. For these reasons, the surgeons would shave these off using a scalpel prior to surgery and have expressed the need for scaffold geometries without sharp protrusions. Hence, the Minor Cavity Fill tool was developed to automatically fill small crevices in the imported bone defect model which would avoid creating such sharp protrusions in the final scaffold geometry.

A completed scaffold design is discussed with the surgeons for their validation along with the bone defect model which is a result of the image segmentation step. The image segmentation step can be a highly technically challenging task, especially when the image has stabilising nails and plates along with bone which often produces metal artefacts on the CT scans. The task is further complicated with suspected sclerotic/necrotic bone. Bone cement in the form of spacers is another material that can exist on CT images but should be removed in surgery. As a result, the segmented 3D bone defect model might possess portions of metal artefacts, sclerotic/necrotic bone and bone cement that is indistinguishable from healthy bone, for which if a scaffold is designed, it would cause problems during surgical implantation. However, if the final scaffold design could be evaluated for their patient-specific fit by comparing them with the CT images which are commonly in DICOM (Digital Imaging and Communications in Medicine) format, rather than the segmented bone defect model, the surgeon would be able to confidently validate the design. Therefore, the DICOM Overlay Registration module was developed to overlay the output scaffold designs on the CT images of the bone defect, which allows the surgeon to compare the scaffold designs directly with the CT images to evaluate its fit and placement, and the overall surgical strategy.

Finally, for clinical validation, the extended workflow was applied to two clinical case studies that were ongoing during its development, a complex bilateral femora defect and a humerus defect, for which scaffolds were designed and successfully surgically implanted. It was also applied to a past clinical case of a defect in the temporal region of a cranium to evaluate its applicability to craniomaxillofacial defects.

## 2 Method: development of the extended features

This section discusses the improvements made to the current workflow and the additional modules developed, followed by the application of the improved workflow to three additional clinical case studies. Please note, the Minor Cavity Fill module is applied to the complex multi-fragmentary femur defect introduced in the previous study Herath et al. (2023) which serves as a clearer example for the tool than the three defect cases mentioned above. In Table 1 the clinical cases are briefly introduced (Human Ethics Exemption Number 20210001814^2^).

**TABLE 1:** Clinical cases for validation of improved workflow.

Clinical case	Anatomical area of the defect	Notes
Case no. 1	Femur (bilateral)	Ongoing clinical case study where the scaffolds were designed and implanted
Case no. 2	Humerus (unilateral)	Ongoing clinical case study where the scaffolds were designed and implanted
Case no. 3	Temporal region of the cranium	A past clinical case used to evaluate the applicability of the workflow to craniomaxillofacial defects
Case no. 4	Femur (unilateral)	The past clinical case used for the demonstration of the original workflow developed in the previous study Herath et al. (2023). This case is used in the current work only to demonstrate the Minor Cavity Fill tool as it serves as a clearer example

### 2.1 Extended features of the improved workflow

Figure 1 illustrates the design workflow with the newly developed modules and improvements. The modules Surgical Resection, Segmental Defect Fill, Minor Cavity Fill, and DICOM Overlay Registration are additions to the current workflow. The algorithm of the Surgical Approach module has been completely redeveloped to take advantage of the F-rep capabilities offered by OpenVDB to improve accuracy and avoid artefacts.

#### 2.1.1 Surgical resection module

**FIGURE 2:**
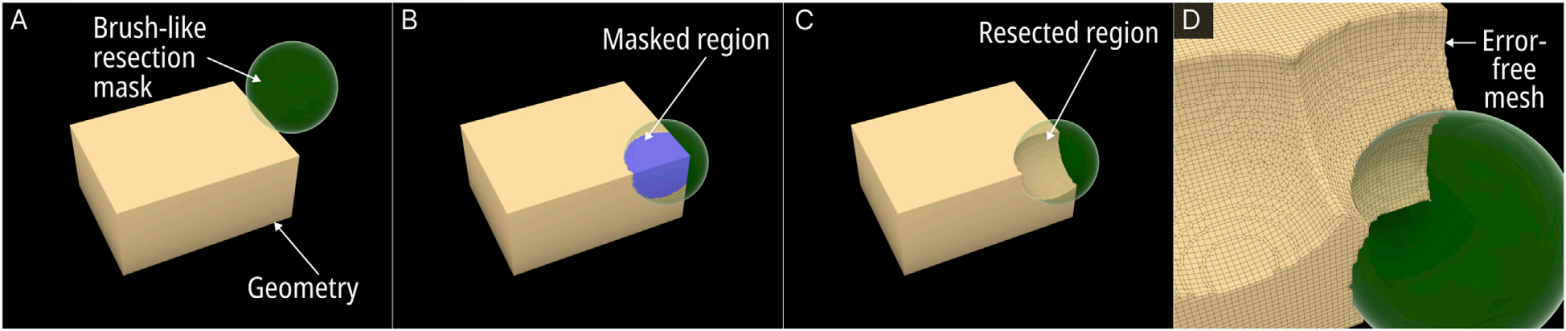
Operational steps of the Surgical Resection module. **(A)** Resection mask (here a sphere). **(B)** Masked region on the Mesh. **(C)** Boolean operation of the masked region and the original mesh creates the resection. **(D)** Close up view of the resected Mesh.

The Surgical Resection module was developed to be a versatile virtual resection tool that allowed the design engineer to quickly remove regions from the bone defect geometry without causing surface mesh errors. Figure 2 displays the steps of the process.

The process involves using a brush-like mask to mark the regions within the viewport which once marked will effectively be removed from the base geometry. An RhGh “Brep” or “Mesh” node assigns any geometry (be it a sphere, cuboid or any other random geometry) that the design engineer wishes to use as a mask. Once assigned, it becomes visible in the viewport as seen in Figure 2A (green spherical mask). A button named “addMask” when clicked activates a custom written C# script which creates a copy of the mask in its current position as a Mesh object within a pre-set layer in RhGh. The script would Boolean union all masks into a single geometry (Equation 1), where “n” is the number of masks on the pre-set layer. This geometry is termed the “resected geometry”.

$$Resected\;geometry=\overset{n}{\underset{i=1}{⋃}}masks_{\left(i\right)}$$ (1)

A Boolean difference between the resected geometry from the input defect geometry provided the post-resected defect geometry (Figure 2C) (Equation 2).

Defectgeometrypostresection=Defectgeometry−Resectedgeometry (2)

All Boolean operations were processed via the F-rep modelling plugin which created error-free output meshes (Figure 2D). Furthermore, every instance the button was clicked, the C# script updated an internal list with the layer’s Mesh objects by referring to their globally unique identifiers (GUIDs). Another button input named ‘undo’ can remove the last created mesh object from the layer by using this list of GUIDs, thus providing an ‘undo’ function to the module. For real-time visualisation of the region of the defect geometry which falls within the current mask, a Boolean intersection operation between the defect geometry and the currently selected mask provided a separate Mesh object which is displayed in the Rhinoceros 3D viewport as can be seen in Figure 2B in blue (Equation 3).

Currentmaskedregion=Defectgeometry∩Currentmask (3)

A virtual surgical resection process involved the designer setting a suitable mask shape to the ‘mask’ node and positioning it on a region that needed to be resected, followed by a click on the “addMask” button. Then the mask object would be moved to a new location and the button pressed again to the newly selected region. The process can be seen in the video in Supplementary Material S1.

Several of these Surgical Resection modules can co-exist in the same workflow. An example of this would be the need to remove foreign (osteosynthesis) material that is either no longer needed or needs to be repositioned (for example, plates, screws or bone cement). Furthermore, it may be necessary to remove portions of bone that are deemed sclerotic/necrotic based on prior surgical experience or available imaging data. The first instance of the module would separate the osteosynthesis material from the imported model, and the second instance would separate the planned-to-be-resected bone from the bone defect model.

#### 2.1.2 Minor cavity fill module

**FIGURE 3:**
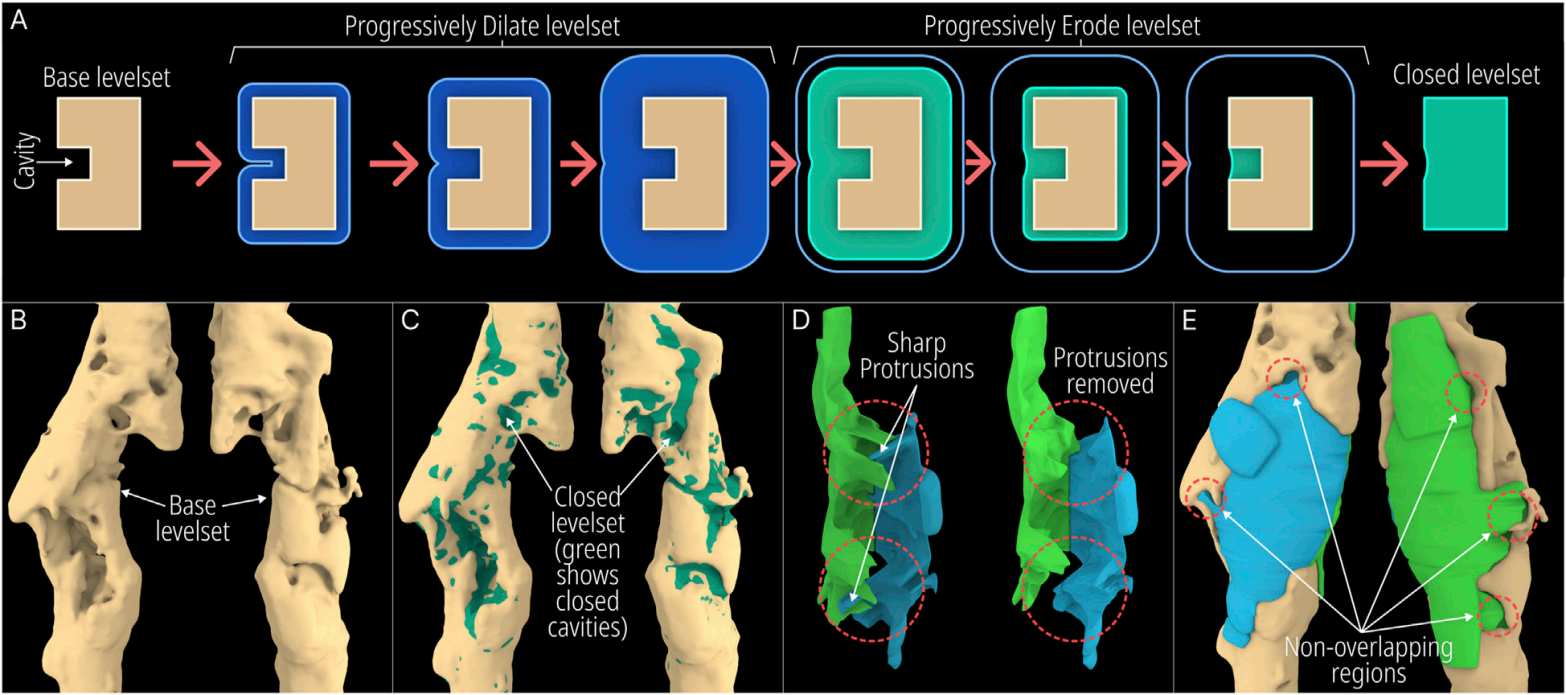
Demonstration of the Minor Cavity Fill module. **(A)** Concept **(B)** base level set with minor cavities, **(C)** closed level set, **(D, E)** resulting differences in scaffold geometries.

This module is implemented entirely within the F-rep domain where the input is a level set Herath et al. (2023). A new tool was introduced to the F-rep plugin that internally uses the OpenVDB filter function ‘offset’ to perform morphological dilation and erosion of level sets progressively. Figure 3A illustrates the concept of closing minor cavities in 2 dimensions (2D) by first dilating (positive offset – seen in blue) the base level set by a pre-set distance which removes the cavity and then eroding (negative offset – seen in green) the dilated level set by the same distance to match the base level set.

The operation applied to the femur defect from the previous study Herath et al. (2023) is seen in Figures 3B, C where in the latter figure, the closed regions can be seen in green. The original and the resulting scaffold designs without the protrusions are seen in Figure 3D. It is evident from the concept that the base geometry remains unaffected. However, as the surface of the bone defect model can have minor troughs, these can be closed off unintentionally. An example of this is seen in Figure 3E where the darker region shows the original scaffolds which are not overlapped by the lighter newly generated scaffolds. These darker patches are minor concavities of the bone defect model which have been closed by the tool.

#### 2.1.3 Segmental defect fill module

**FIGURE 4:**
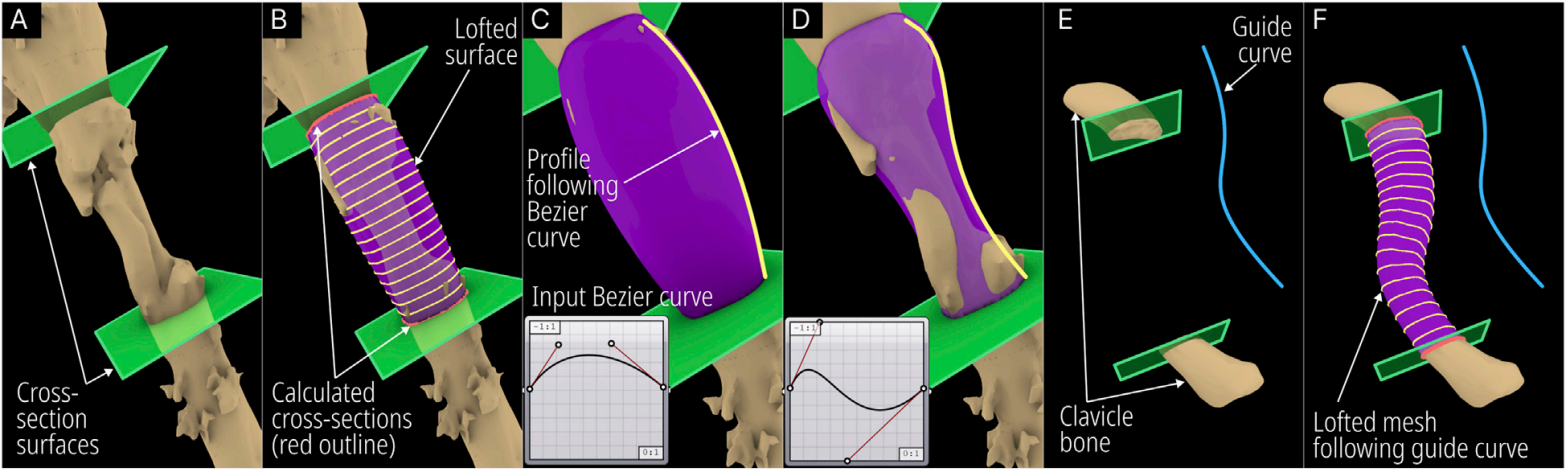
Operational steps of the Segmental Defect Fill module. **(A)** User positioned cross-sections (green planes). **(B)** Intersection between cross-section and bone defect model (red) and lofted interpolation lines (yellow) following a Bezier curve. **(C)** Default Bezier curve profile. **(D)** Modified Bezier curve profile. **(E)** User defined guide curve to bridge defect and **(F)** calculated interpolated mesh following the guide curve.

The Segmental Defect Fill module was developed to create an interpolated surface between two user-selected cross-sections of the bone, which makes it suitable for segmental defects. The process is illustrated in Figure 4.

The module was developed to accept the bone defect mesh, two user-created surfaces, a Bezier curve, and an optional guide curve as inputs. The design engineer needs to create two surfaces within Rhinoceros 3D at the cross-sections that are needed to be bridged and a lofted surface mesh is automatically created by the module. Figure 4A shows the humerus defect (case 2) and the two surfaces created on the either side of the defected region. The module calculates the closed intersection curves (red – Figure 4B) between the surfaces and the defect mesh, and closed curves are interpolated (yellow - Figure 4B) between them according to the Bezier curve inputs. The Bezier curve input allows the user to manipulate the profile of the lofted geometry as can be seen in Figures 4C, D. Furthermore, it can be made to follow a user-created guide curve as seen in the case of a defected clavicle shown in Figures 4E, F.

#### 2.1.4 Improved surgical approach module

**FIGURE 5:**
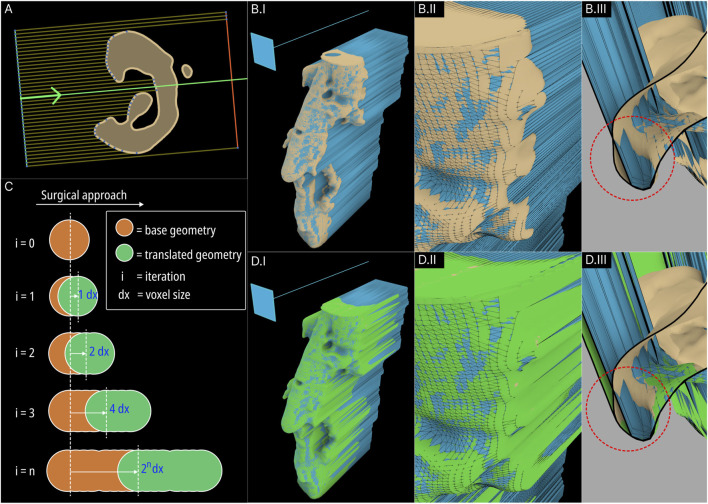
Concept of the original B-rep based Surgical Approach module (resultant projected geometry is coloured blue) **(A)** and its artefacts **(B)**. The concept of the new F-rep based module **(C)** and its resulting projected level set (coloured green) **(D)**.

The original Surgical Approach module was developed in the previous study Herath et al. (2023) to avoid any obstructions to scaffold insertion by using a projected mesh for its primary Boolean operations. Figures 5A, B illustrates the femur defect case (case 4) as seen in the previous study Herath et al. (2023) depicting the B-rep based method followed by the original Surgical Approach module to create the projected mesh. As illustrated in Figure 5A, the original module functioned by projecting rays from a mesh-based surface placed in the front of the defect geometry, through the defect, towards a mesh surface placed behind it, and creating a surface based on the ray collisions. However, if the resolution of the mesh surfaces is insufficient, artefacts can be produced as seen in Figure 5B, which are the result of elongated triangles whose vertices are on the defect geometry as well as on the mesh surface behind.

To avoid these artefacts, the surgical approach module was redeveloped using F-rep modelling. The projected mesh is the result of geometric sweeping of the defect mesh towards the surgical approach direction. This was performed by iteratively translating the level set towards a vector in-line with the surgical approach direction incrementally and combining them using a Boolean union operation in every iteration. This mechanism is illustrated in Figure 5C. As the surgical approach vector does not vary for all iterations, to reduce computational overhead, in each iteration, the combined level set from the previous iteration is used and the translating distance is compounded. The concept is illustrated in Figure 5C. In the first iteration, the translated distance is the voxel size of the OpenVDB grid the level set is stored in. The second iteration will translate the now-combined geometry from the first iteration by 2 voxel sizes. This process is iterated until the projected geometry reaches the required sweeping length which is a factor of the length of the bone defect mesh towards the surgical approach direction.

Figure 5D shows the new swept (projected) geometry in green overlaid on the B-rep based result. The artefacts and deformities in the B-rep based projected geometry (seen in Figure 5BII by the beige-coloured base geometry protruding out of the blue projected geometry) have now been covered by the F-rep based projected geometry. Figure 5BIII and Figure 5DIII show close ups of cross-section of the resulting geometries which clearly shows the F-rep geometry’s accuracy of conforming to the edges of the base geometry.

#### 2.1.5 DICOM Overlay Registration module

**FIGURE 6:**
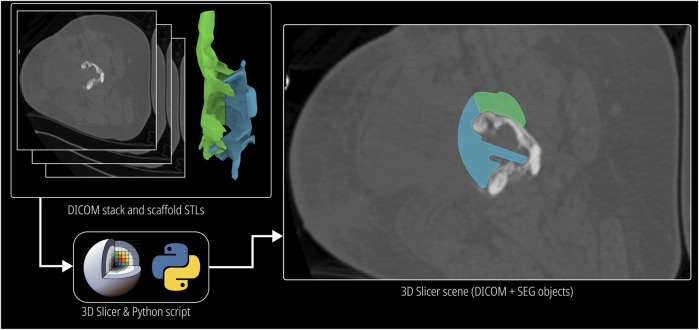
Process and demonstration of the DICOM Overlay Registration module.

This module was developed to compare the scaffold designs with the CT image data of the patient which are stored in DICOM format. The open source image segmentation software 3D Slicer Kikinis et al. (2014) was used to read and visualise DICOM data. As most segmenting software uses the same coordinate space to export the surface meshes of the segmented 3D models, the origin when imported to Rhinoceros 3D (the workflow does not alter the orientation of the imported defect model) as well as the origin of all exported scaffold designs are preserved. Hence, when they are imported as 3D models in 3D Slicer, they appear as a transparent overlay which has its own layer colour. 3D Slicer allows the overlay including the volumetric CT data be embedded into a “scene” file which can be shared with the surgeon for scaffold design validation. The respective scaffold image slices will be seen on each image of the DICOM image stack, through which the surgeon can conveniently scroll and analyse scaffold design and fit. However, this approach relies on the surgeon’s familiarity of the 3D Slicer software. This method is visualised in Figure 6.

An alternative approach was developed to avoid this constraint by embedding a grey scale mask of the scaffold on to the image slices and creating a new DICOM image stack, which can be sent to the surgeon to be viewed in their preferred DICOM viewer, thus not restricting the surgeon to install and familiarise themselves with 3D Slicer. A python script uses the application programming interface (API) of 3D Slicer to isolate the voxels which coincide with scaffold models and increase their intensity by a pre-set value to create a greyscale mask which demarcates the scaffold on top of the DICOM images while retaining the actual image data within the mask. The inputs to the python script are the surface meshes of the scaffold models and the DICOM image stack of the bone defect. Its output is a new anonymised DICOM image stack with the greyscale mask embedded in it. The greyscale output of this method can be seen in Supplementary Figure S7.

The modules of the extended workflow and their functions are summarised in Table 2. The modules that were added in the course of this research are demarcated.

**TABLE 2:** The modules of the extended workflow and their functions. The new and improved modules developed in the course of this research are marked with an asterisk (*).

Group	Module	Function
Import	Defect mesh import	Import the bone defect surface mesh
	ROI isolation	Create the ROI mesh which isolates the region of interest from the imported bone defect mesh
Scaffold Geometry Creation	Surgical resection (new)*	Virtual resection tool that allowed the design engineer to quickly remove regions from the bone defect geometry without causing surface mesh errors
	Minor cavity fill (new)*	Produces scaffold designs with smooth external surfaces removing sharp protrusions caused by patient-specific fitting to narrow defect cavities
	Defect cavity fill	Creates a envelope geometry by filling the cavities via a intersecting planes. This is preferable for trauma defects
	Guided fill	Manipulate the cavity fill via guide user-created guide curves
	Segmental defect fill (new)*	Convenient envelope shape generation for segmental defects with profile control and optional guiding capability
	Surgical approach (improved)*	Ensures the patient-specific fit and unobstructive insertion from the planned surgical approach. This module was redeveloped from the B-rep based method to an F-rep based one to remove resolution dependent artefacts
	Fixation flanges	Boolean unions the flange geometries to the solid scaffold geometry and create a plane to demarcate the flanges from the scaffold body that is to be made porous
Porous Architectures	Voronoi	Converts the solid scaffold geometry into a Voronoi tessellation based porous scaffold geometry
	Periodic lattice	Converts the solid scaffold geometry into a periodic lattice (simple cubic, body centred cubic, face centred cubic) based porous scaffold geometry
	TPMS	Converts the solid scaffold geometry into a TPMS (e.g. Gyroid, Neovius, Schwarz) based porous scaffold geometry
Export	Export surface mesh	Converts the level sets into triangular surface meshes (either adaptive or not) and exports them out of RhGh
Scaffold Evaulation	Porosity calculation	Calculates the porosity of the scaffold design
	Pore size distribution	Calculates the distribution of pore diameters
	Virtual scaffold insertion	Virtually examines cross-sections of the model during the insertion of the scaffolds into the bone defect
	3D printability check	Checks the output surface mesh for mesh errors (self-intersected faces, degenerated faces, manifold edges and if the mesh is closed/watertight)
	DICOM overlay registration (new)*	Overlay the scaffold designs on the DICOM image stack of the defect for design verification by the surgeon

### 2.2 Application of improved workflow to clinical case studies

To evaluate the applicability of the improved workflow to a diverse range of clinical cases, it was applied to three additional clinical case studies, namely a bilateral femur defect (case 1), a humerus defect (case 2), and craniomaxillofacial defect (case 3). The first two cases took place during the development of the workflow and hence, scaffolds were designed using the workflow, manufactured by a designated manufacturer, and were surgically implanted by the team of surgeons. The craniomaxillofacial defect is an exemplar case study used to demonstrate the applicability of the workflow to craniomaxillofacial defects.

Following the generation of scaffold geometries, they were inspected for the presence of 3D printing mesh errors, namely, being free from degenerate or self-intersecting faces, is watertight and manifold using RhGh as well as the commercial-grade slicing and GCode generation software Simplify3D (Simplify3D, Ohio, United States).

Then their patient-specific fit and unobstructive insertion were evaluated firstly using digital inspection of their random cross-sections, secondly, by 3D printing prototypes of the defect and the generated scaffolds and inserting the scaffold pieces into the defect, and thirdly, using the DICOM Overlay Registration tool. The 3D printing was carried out using polylactic acid (PLA) with the 3D printer Flashforge Dreamer (Zhejiang Flashforge 3D Technology Co., Ltd., Zhejiang Province, China) using a layer height of 0.18 mm, nozzle diameter of 0.4 mm, print speed of 30 mm/s, nozzle temperature of 205°C, build temperature of 60°C and with support where necessary for all models.

Once the surgical strategy and its respective designs of the bilateral-femora and a humerus cases were finalised, the solid scaffold geometries were sent to the designated manufacturer to be 3D printed under sterilised conditions using the medical-grade bioresorbable polymer polycaprolactone and tricalcium phosphate (mPCL-TCP). The solid geometries were sent because the manufacturer was only able to fabricate scaffolds with a sparse rectilinear infill (alternating layers of 0°, 60°,120° degrees) architecture. Once manufactured, they were surgically implanted by the team of surgeons.

## 3 Results: clinical application and evaluation

The developed workflow applied to three clinical cases, a case of bilateral-femora defect (case 1), a case of a humerus defect (case 2), and a case of a craniomaxillofacial defect (case 3), are discussed in this section.

### 3.1 Workflow applied to the clinical case studies

#### 3.1.1 Case 1: bilateral femoral defect

The patient presented to the Department of Orthopaedics, Trauma and Reconstructive Surgery of the University Hospital RWTH Aachen with bilateral femoral non-unions with significant bone defects after failed (ex domo) treatment of bilateral distal femur fractures. In the right femur, radiological diagnosis revealed a plate inserted from the lateral side, with no evidence of screw loosening. It was therefore decided not to remove the inserted osteosynthesis material in the forthcoming surgical procedure and in fact to use the inserted screws as landmarks to define the cutting planes for removal of sclerotic and necrotic bone (Supplementary Figure S1). In the area of the left distal femur, there was medial and lateral insertion of one plate each. Overall, the inserted plates and screws were considered stable with no signs of implant loosening (Supplementary Figure S2). However, closer radiological examination and further interdisciplinary discussion revealed the need to remove three of the inserted screws. Two of the three screws were in the area of the bone defect and therefore had no stabilizing function there, and the third screw had broken off, which required its removal (Supplementary Figure S3). The analysis of the 3D reconstruction of the bone defect in the area of the left distal femur showed the necessity of the removal of sclerosed and necrotic bone (Supplementary Figure S4). This facilitates the implantability on the one hand and guarantees the scaffold implantation in a vital wound bed on the other hand which facilitates the ingrowth and the bone regeneration.

**FIGURE 7:**
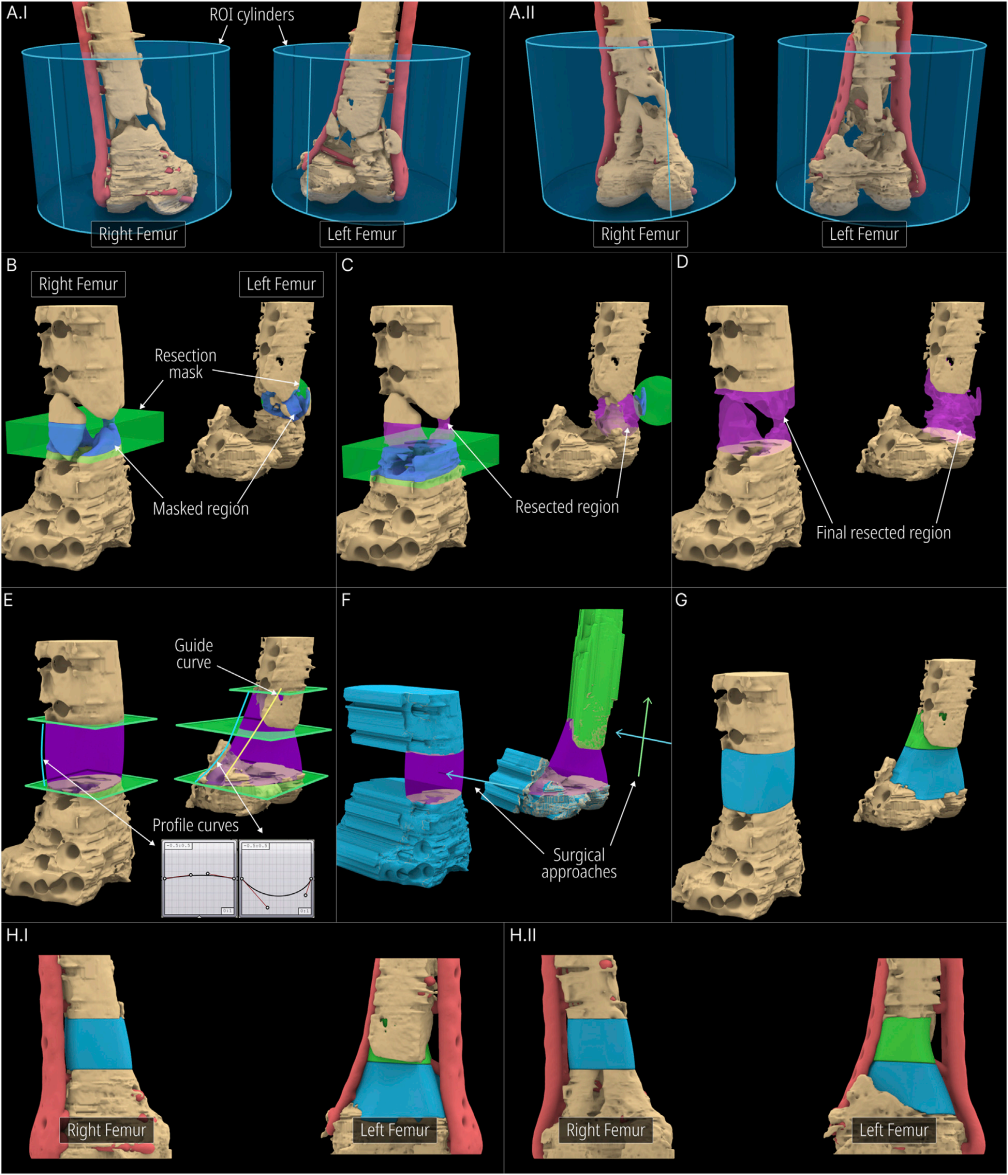
Application of workflow to the bilateral-femora defect. **(A)** ROI isolation. **(B–D)** Surgical resection procedure. **(E)** Cavity filled geometries created using the Segmental Defect Fill module. **(F, G)** Level-sets created using the Improved Surgical Approach module. **(H)** Final scaffold designs.

The workflow applied to the case is visualised in Figure 7. Both femora were segmented as a single 3D model and was imported into RhGh. The stabilising plate and the screws were segmented separately and was imported as a separate model. Both models can be seen in Figure 7I. The region of interest (ROI) was isolated using an ROI Cylinder which is the first step of the Scaffold Geometry Creation as seen in Figure 1. Once the ROI was isolated, using the Virtual Resection Tool, the necrotic and planned-to-be-resected bone was removed. A rectangular mask (Figure 7B) was suitable for the right femur where the resection was performed using a straight edge cutting saw. However, for the left femur, a spherical mask (Figure 7B) provided easier resection as much of the sclerotic/necrotic bone was irregular in shape. The figures Figures 7B–D illustrates the resection process including the masks (green), masked regions (blue), resected regions (purple) and lastly the final resected geometries (beige). Similarly, the dislocated and broken screws which were a part of the screws and plate model that was imported earlier were also removed using the Virtual Resection Tool.

Once the resection is complete, three segmenting planes were positioned as shown in Figure 7E to generate three scaffold pieces and they were assigned to the Segmental Fill module. The two Bezier curves were used to control the profile curves which affects the shape of the segment fill shape (purple). Furthermore, a guide curve was used for the left femur for better control over the shape. The general surgical approach was decided to be from the anterior direction. However, the top scaffold piece for the left femur was planned to be inserted from the anterior direction and lifted cranially into the cavity. Therefore, its surgical approach was set in a caudal-to-cranial direction as seen by the green arrow in Figure 7F. The blue arrows indicate the surgical approaches for the other two scaffold pieces. The resulting projected geometries created by the improved surgical approach module are seen in Figure 7F. The solid scaffold geometries which are generated through the Boolean operations between the projected geometries, the segmental fill geometry and the screws and plate geometry are shown in Figure 7G. The final solid scaffold geometries as seen from the anterior and posterior views are illustrated in Figure 7HI-II.

The solid scaffold geometries were not converted into porous scaffolds as the designated manufacturer was only able to fabricate scaffolds with the sparse rectilinear infill. Therefore, the solid scaffold geometries were exported and sent to the manufacturer. The manufactured scaffolds are pictured in Supplementary Figure S6.

#### 3.1.2 Case 2: humerus defect

**FIGURE 8:**
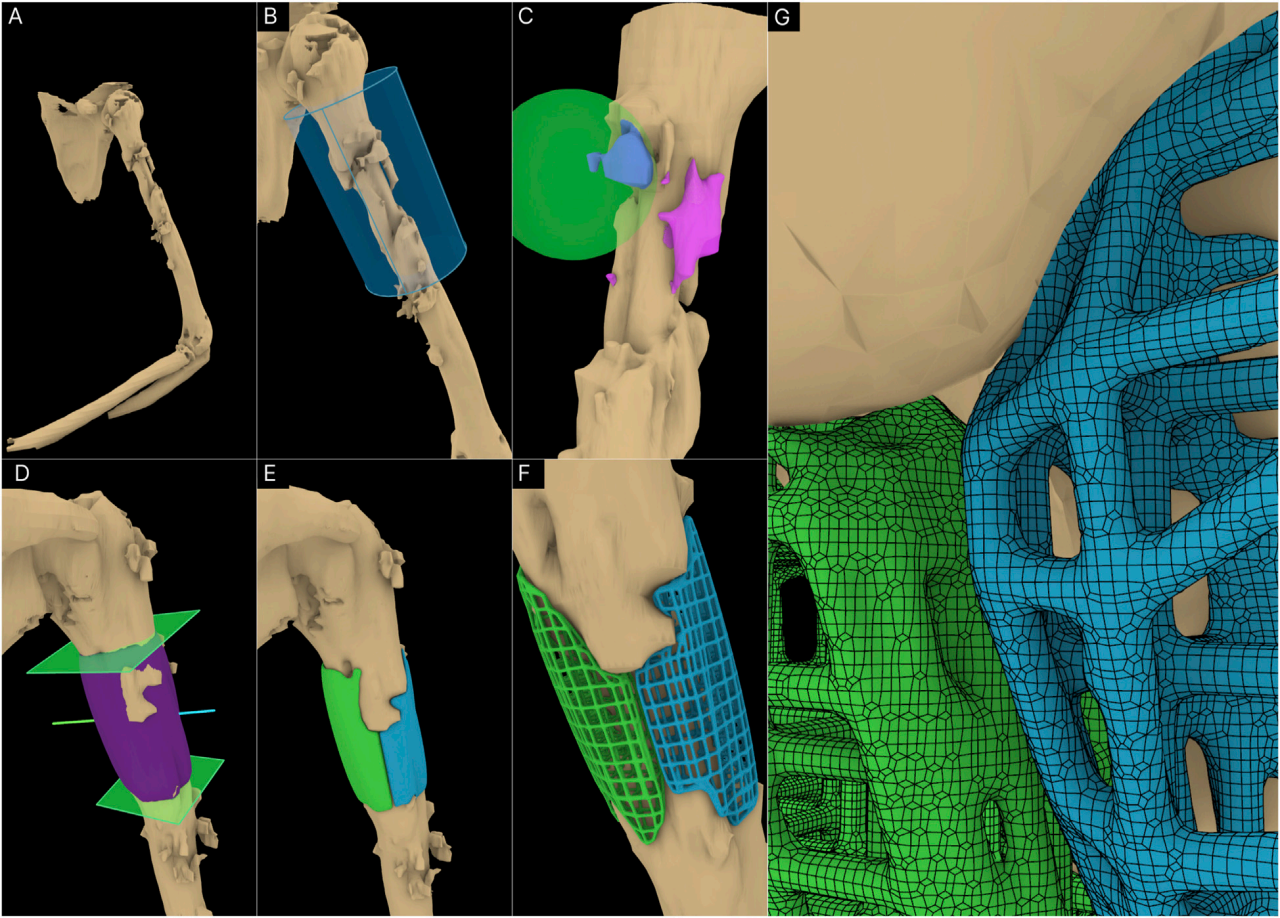
Application of workflow to the humerus defect. **(A)** 3D defect model of the humerus. **(B)** ROI Isolation. **(C)** Virtual resection of bone protrusions. **(D)** Cavity filled geometry created using Segmental Fill Module. The surgical approaches used (blue and green lines) can also be seen. **(E)** Solid scaffold geometries generated. **(F)** Porous scaffold geometries. **(G)** Surface mesh of the porous scaffold geometries.

After initial ex domo treatment of a left proximal humeral shaft fracture by insertion of an intramedullary nail, the healing of the fracture failed and the patient presented to the Department of Orthopaedics, Trauma and Reconstructive Surgery of the University Hospital RWTH Aachen. Briefly, a larger bone defect was found for which the clinical team also aimed at bone regeneration by implantation of 3D printed scaffolds. The rationale for a modular scaffold in this case was also to leave the inlaying osteosynthesis material, i.e. the intramedullary nail, in place, as there was no signs of implant loosening. Accordingly, the aim was to perform a scaffold implantation in order to achieve bone regeneration and thus achieve better biomechanical stability in the humerus. Therefore, using the design workflow, two scaffold halves were created. The 3D surface mesh of the humerus was imported following image segmentation into RhGh and an ROI cylinder was positioned over the relevant region (Figures 8.A, B).

The intramedullary nail was included in the imported bone defect model. It was left as it is as it would not be an issue with the scaffold generation process. The Surgical Resection tool was used to remove a few bone protrusions the surgeons decided should be resected (Figure 8C). Two segmental planes were positioned on either end of the defect for the Segmental Defect Fill module as seen in Figure 8D. This figure also displays the surgical approaches set for the two scaffold halves. The solid scaffold geometries can be seen in Figure 8E followed by Figure 8F where they were converted to periodic lattice-based scaffolds. Figure 8G displays the surface mesh composed of quads and triangles created by the F-rep plugin.

However, the designated manufacturer could only 3D print the solid scaffold geometries with the rectilinear infill (alternating layers of 0°, 60°, 120° degrees) architecture given their 3D printing capabilities. These were 3D printed and sent to the team of surgeons where they were implanted. The manufactured scaffolds are pictured in Supplementary Figure S6.

### 3.1.3 Case 3: craniomaxillofacial defect

**FIGURE 9:**
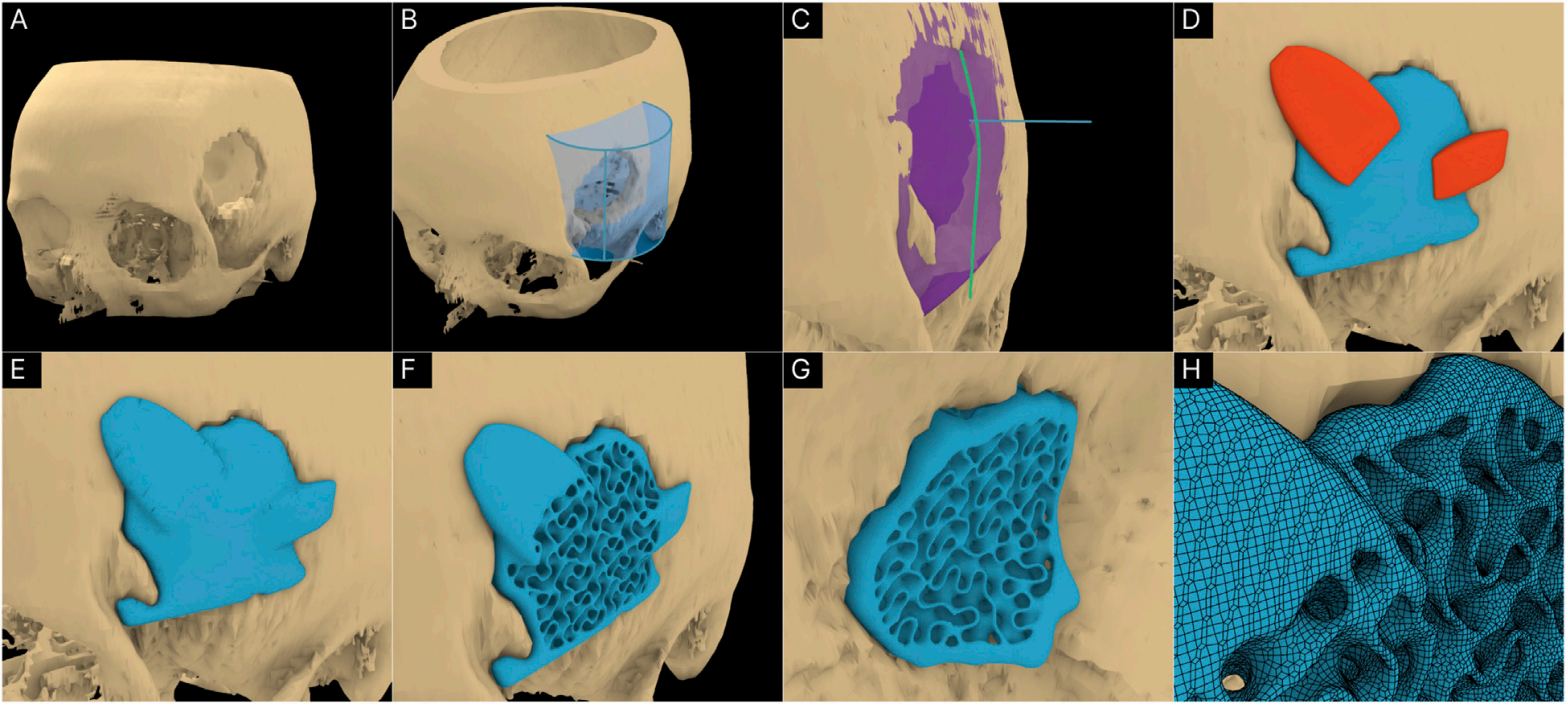
Application of workflow to the craniomaxillofacial defect. **(A)** 3D model of the cranium including the defect. **(B)** ROI isolation. **(C)** Guided Cavity Fill module and setting of the surgical approach (straight blue line segment). **(D, E)** Adding fixation flanges to the scaffold geometry. **(F, G)** Porous scaffold geometry based on the Gyroid TPMS structure. **(H)** Surface mesh of the porous scaffold geometry.

A craniomaxillofacial defect was used as an example case to further test the workflow’s range of applicability. Figures 9A, B shows the defect cavity in the temporal region of the cranium and the ROI cylinder positioned over this region. The Guided Cavity Fill module was used with the guide curve (green) seen in Figure 9C. The surgical approach was set orthogonally to the surface as seen by the blue line in Figure 9C. Once the solid scaffold was generated, two flanges were created using the Fixation Flanges module and was automatically merged to the scaffold (Figure 9D). Finally, the solid scaffold (Figure 9E) was converted into a porous scaffold geometry based on the Gyroid triply periodic minimal surface (TPMS) based architecture (Figures 9F, G). Figure 9H displays the surface mesh generated by the F-rep plugin composed of quads and triangles.

### 3.2 3D printability check

The designed scaffolds were checked for 3D printing mesh errors that obstruct the slicing and GCode generation process of 3D printing. The checks were done on RhGh as well as on Simplify3D. The results are tabulated in Table 3.

**TABLE 3:** Checking the scaffold design meshes for mesh errors using RhGh and Simplyfy3D (S3D). Sol:solid; Por:porous; R:right; L:left; t:top; b:bottom; A:part A; B:part B; Gy:gyroid.

Mesh check	Bilateral-femora			Humerus				Cranium	
	SolR	SolLt	SolLb	SolA	SolB	PorA	PorB	SolA	PorGy
RhGh - Non-manifold edges	Pass	Pass	Pass	Pass	Pass	Pass	Pass	Pass	Pass
RhGh - Self-intersecting faces	Pass	Pass	Pass	Pass	Pass	Pass	Pass	Pass	Pass
RhGh - Naked edges	Pass	Pass	Pass	Pass	Pass	Pass	Pass	Pass	Pass
RhGh - Duplicated faces	Pass	Pass	Pass	Pass	Pass	Pass	Pass	Pass	Pass
RhGh - Degenerate faces	Pass	Pass	Pass	Pass	Pass	Pass	Pass	Pass	Pass
S3D - Non-manifold edges	Pass	Pass	Pass	Pass	Pass	Pass	Pass	Pass	Pass
S3D - Self-intersecting faces	Pass	Pass	Pass	Pass	Pass	Pass	Pass	Pass	Pass
S3D - Duplicated faces	Pass	Pass	Pass	Pass	Pass	Pass	Pass	Pass	Pass
S3D - Degenerate faces	Pass	Pass	Pass	Pass	Pass	Pass	Pass	Pass	Pass

With the help of the F-rep plugin, all models produced by the workflow was error-free and readily sliceable in Simplify3D without requiring any post-processing.

### 3.3 Evaluation of patient-specific fit and unobstructive surgical insertion

**FIGURE 10:**
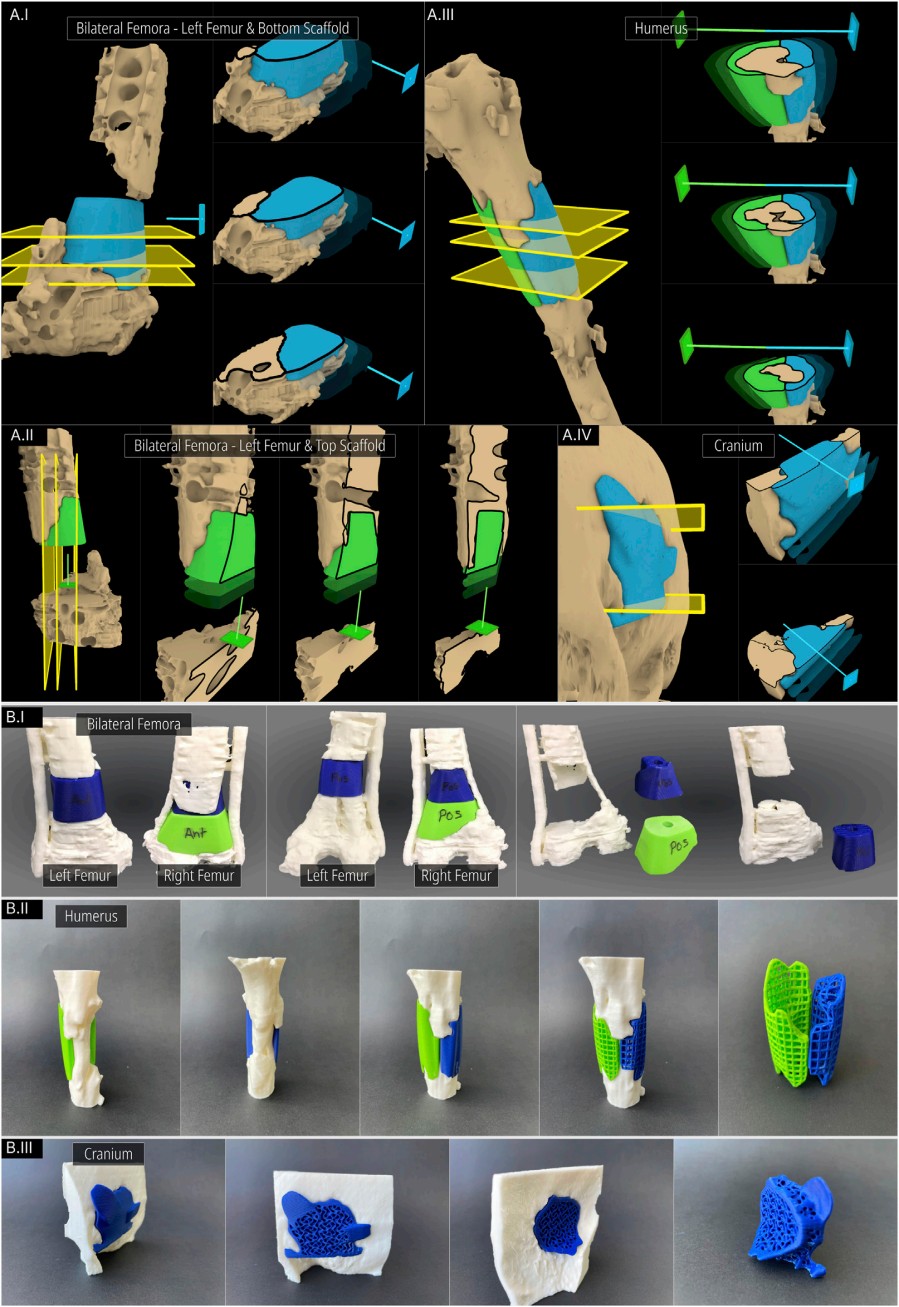
Inspecting the patient-specific fit and unobstructive insertion. **(A)** Digital inspection of random cross sections. **(B)** 3D printed prototypes of the defects and their scaffolds.

Digital inspection of three random cross sections of the defects and the solid scaffolds are displayed in Figure 10 of the three cases, the bilateral femora (case 1), humerus (case 2) and the craniomaxillofacial (case 3) defects. All scaffolds are depicted in three instances of their insertion from the respective surgical approach with increasing opacities.

In Figure 10AI and Figure 10AII the screws and plates of the bilateral femora were hidden for visual clarity. The right femur is not included in Figure 10A as the segments were cleanly cut with the straight edged saw which created a scaffold that had planar surfaces. This ensures patient-specificity on its own and a cross-sectional view in the plane orthogonal to the surgical approach direction is not required as there is no bone between the segmental planes to obstruct its insertion. In Figure 10AII which shows the left femur of the bilateral femora case, as the surgical approach was set to the caudal-to-cranial direction for the top scaffold, the cross sections are shown vertically. The unobstructive insertion of all scaffolds as well as an ideal patient-specific fit are evident from these random cross section illustrations.

Figure 10B shows the 3D printed solid and porous scaffold geometries of all three defect cases. All scaffolds were designed with a clearance of 0.1 mm with the defect model which can be set in the workflow. The insertion of all scaffolds was effortless and posed no obstructions. Figure 10B shows the solid scaffolds of the bilateral femora, both inserted into the defect, and independently where cylindrical holes can be seen in both which were used to install a bone morphogenetic protein (BMP) filled collagen sponge to enhance bone regeneration. Figure 10BII displays the solid scaffolds individually inserted into the humerus defect cavity where an ideal patient-specific fit can be seen. It also shows both solid scaffolds inserted into the cavity, showing a good seal between bone and the two-halves of the scaffolds. Lastly, the porous scaffolds based on a periodic lattice architecture are inserted into the cavity, which show the same patient-specific fit as the solid scaffolds. The outer contouring edges which are automatically generated by the Period Lattice module of the Scaffold Architectures section of the workflow can be seen on these two porous scaffolds. Figure 10BIII shows the solid scaffold inserted into the craniomaxillofacial defect followed by its porous version. The inside of the defect cavity is visible where an ideal patient-specific fit can be observed. Lastly, it shows the independent scaffold based on the Gyroid TPMS surface, along with the feature edges of the scaffold which are automatically generated by the TPMS module of the Scaffold Architectures section.

**FIGURE 11:**
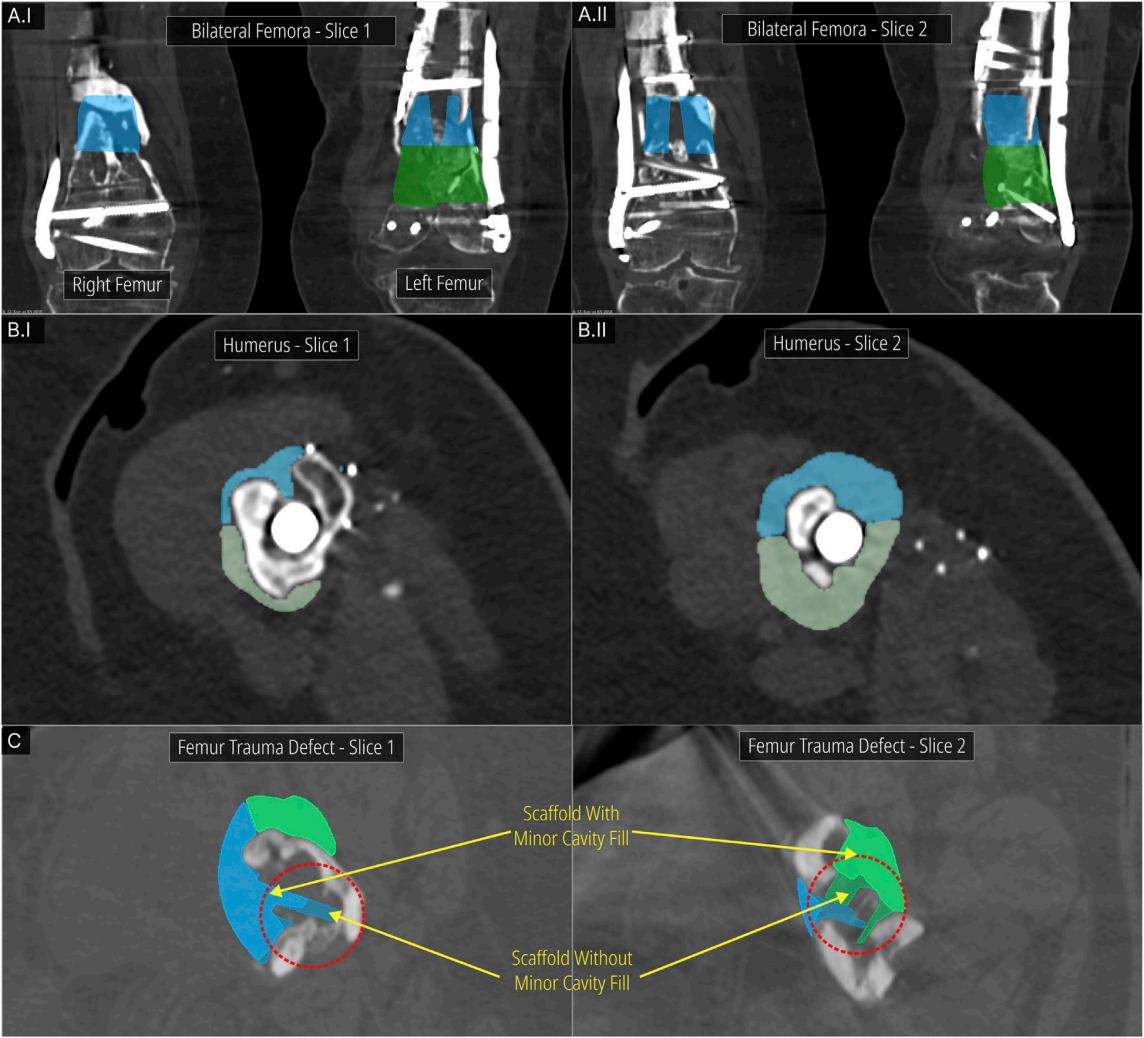
DICOM Overlay Registration module. **(A)** Bilateral femora defect case. **(B)** Humerus defect case. **(C)** Effect of the Minor Cavity Fill tool on the femoral trauma defect from the previous study.

Using the DICOM Overlay Registration module, the scaffold geometries were overlaid on the DICOM images using the python script and 3D Slicer where overlays for each scaffold is generated with reduced opacity. Figure 11 shows the application of the module to the bilateral femora (case 1), humerus (case 2) and the multi-fragmentary femur defect (case 4) cases.

Figure 11A show two DICOM slices of the bilateral femora defect (case 1). The bone regions that were to be resected and the screws that were to be removed are seen overlapping with the scaffold overlays. Figure 11B display two DICOM slices of the humerus defect (case 2) and its scaffold halves. Again, the overlapping bone region is to be resected. Figure 11C shows two DICOM slices for the femoral trauma defect which also shows the result of using the Minor Cavity Fill tool. Two sets of scaffolds created with and without the tool are shown overlapped with one another which clearly shows the lack of sharp protrusions in the scaffolds created by using the tool. The patient-specific fit is not compromised as a result of the Minor Cavity Fill tool as seen in these figures.

### 3.4 Surgical implantation

With the bilateral femora defect (case 1), the scaffolds were loaded with autologous bone graft according to the SGBR concept Laubach et al. (2023) and inserted within one operation. For this purpose, on both sides, a lateral skin incision was made on the femur, with the defect area being approached from the antero-lateral direction. According to the preoperative planning of the cutting planes, sclerotic and necrotic bone on the right femur (Supplementary Figure S1) as well as the corresponding bone portions on the left femur (Supplementary Figure S4) could be removed without any problems and the adjacent bone margins refreshed. During implantation, it became apparent that the scaffold circumference was discreetly too large (<5 mm) as the manufacturer 3D printed the scaffolds at a slightly larger scale to compensate for expected temperature induced shrinkage which was communicated by them to the team of surgeons. After appropriate minimal (<5 mm) resection of the edges of the scaffold, an implantation without complications was achieved. In particular, a smooth modular implantation was performed, with the cranial scaffold first being inserted from the caudal direction and then pushed upwards. The second scaffold was then inserted anteriorly without complications and a sufficient filling of the defect was achieved. Subsequently, a layer-by-layer wound closure without complications could be performed.

For the humerus defect (case 2), the skin incision was made through an antero-lateral approach on the left upper arm. After careful dissection into the depth, the exposed nail in the area of the proximal humerus was exposed. According to the preoperative interdisciplinary planning, a careful attempt was made to fit the two-piece modular scaffold. However, the medial region showed exposed neurovascular structures that might be injured by the insertion of the scaffold. Therefore, it was decided intraoperatively not to insert the medial part of the modular scaffold. In the lateral region, a scaffold loaded with autologous bone material was placed as planned without any difficulties (Supplementary Figure S5). In addition, autologous bone material was inserted in the medial region to achieve bone regeneration. Subsequently, the wound was closed in layers without complications.

## 4 Discussion

Clinical studies have effectively concluded the efficacy of patient-specific SGBR scaffolds for treating even complex bone defects Laubach et al. (2023). A bottom-up patient-specific scaffold design workflow was developed and applied to a complex trauma defect Herath et al. (2023). In this study, the workflow was extended with additional features to virtually resect geometric regions where needed, automatically fill minor defect cavities to prevent sharp protrusions being generated on the scaffolds, fill segmental defect cavities, and evaluate scaffold designs overlaid on the CT images. Furthermore, the surgical approach module was redeveloped to adopt an F-rep approach which avoids resolution-based artefacts commonly seen on scaffolds generated by its predecessor. The extended workflow was applied to three clinical case studies, a defect of bilateral femora, a humerus defect and a craniomaxillofacial defect. The first two clinical cases took place during the development of the workflow and hence, scaffolds were designed appropriately using the workflow and were subsequently manufactured and surgically implanted successfully. The cranium defect was used as an exemplar to gauge the applicability of the workflow to a broader range of defects.

The Surgical Resection tool proved a versatile method to quickly remove regions of any geometric model using the implicit geometry (F-rep) domain, which assures mesh-error free output models. This tool was effectively used in both defect cases, the bilateral femora and the humerus, to remove sclerotic/necrotic bone, healthy bone segments that is planned to be resected prior to scaffold insertion, and also broken screws which were to be removed that were parts of the image segmented model belonging to the stabilising plate and screws. The Minor Cavity Fill tool which was demonstrated on the femur defect used in the previous study, was able to close small cavities in the defect model often created from trauma or improper bone regeneration, which resulted in smooth scaffold geometries free from sharp protrusions. This was confirmed using the DICOM Overlay Registration module as was seen in Figure 11C. Finally, the masks that are continuously created by clicking the ‘addMask’ button are locally stored independently on the designated layer within RhGh. Therefore, the entire virtual resection process is non-destructive in nature and can be easily reversed using the ‘undo’ button at any point in time during the scaffold designing process.

The Segmental Defect Fill module was able to successfully create defect cavity fills for both the bilateral femora defects and the humerus defect. The Bezier profile curve and the optional guide curve grants design flexibility to address a broad range segmental defects. All the inputs except for the segmental planes demarcating the either ends of the defect cavity are numerical, and the surgical strategy governs the placement of these segmental planes. As a result, the repeatability and objectivity of this procedure is not compromised compared to manual geometric procedures such as sculpting.

The redeveloped Surgical Approach module was able to replicate the projected geometry towards the surgical direction selected with ideal accuracy as seen from Figure 5DII. This ensures that there will be no artefacts which could create slight obstructions near the edges of the defect geometry provided the resolution is insufficient. The improved module uses the same voxel size as the one used in the initial conversion of the input bone defect to a level set. Hence, the resolutions of the geometries are seamless with one another, and are governed by the initial voxel size which is a single input to the entire workflow. Furthermore, in the grander plan to devise a bottom-up standalone software tool powered by F-rep, the improved module which now uses OpenVDB internally can directly be used, avoiding the need to create a B-rep modelling approach.

As 3D models are the product of image segmentation, metallic flares or artefacts can easily create apparent bone. Moreover, compared to the use of CT image stack data, important limitations are reported for 3D prints, which relate to the lack of information on bone quality and on artefacts such as implants, their presence and position (e.g. broken screws in the bone) Popescu et al. (2021). Surgeons are trained to interpret and navigate through CT image stacks, making them a preferred choice in a clinical setting Wesorick et al. (2024). Therefore, when seeking consensus from the surgeons on a scaffold design, we learnt from our team of surgeons that it would greatly support them to view the scaffold designs overlaid on the CT images, which is the purpose of the DICOM Overlay Registration module. It allows the direct comparison of the output scaffold designs against the ground truth that is the CT images of the defect. Therefore, the surgeon can validate and confirm that the design is suitable to be used based on inspecting the overlay of the scaffold design over the DICOM image stack. Lastly, the design iterations of the scaffold design process could drastically reduce as surgeons could validate the design prior to 3D printing prototypes.

All scaffold designs of the three defect cases proved to have patient-specific fit using all three methods, via 3D printed prototypes, virtual cross-sections and digital insertion and lastly, using the DICOM Overlay Registration tool. The F-rep plugin previously developed for the workflow was able to successfully create surface meshes of all level sets without any surface mesh errors that could prevent the slicing of these models (Table 3). Furthermore, taking into account recent developments in the field of orthopaedic regenerative medicine for large bone defects Jia et al. (2023), we were able to design the scaffolds for case 1 (Figure 10B) in such a way that a central cylindrical hole could be equipped with a BMP-eluting collagen sponge. Sustained release of BMP is known for its ability to successfully regenerate bone Henkel et al. (2021), and the scaffold design, which allows for a protected BMP-elluting collagen sponge, has been associated with improved bone regeneration Yang et al. (2021).

There are several limitations inherent to the workflow which still remain valid. Although envisioned to be used by the surgeons themselves, the current version of the workflow lacks a dedicated graphical user interface (GUI) which can guide a surgeon to design a scaffold by themselves. Furthermore, the workflow is still reliant on a design engineer who is familiar with the fundamentals of the RhGh software suite.

The workflow developed is expected to be progressively improved in three avenues. With increasing number of functions being moved to the F-rep realm, the porting of the workflow principles to its own dedicated software platform that is running on an F-rep modelling kernel is achievable and is currently explored by our research group. Additionally, the mechanical properties of a SGBR scaffold are an important aspect stemming from its design. A scaffold design should withstand not only the everyday biomechanical loading forces it is exposed to even if exposed partially in the case of being implanted alongside an intramedullary nail or stabilising plate, but it should also withstand intraoperative loading forces, which can sometimes be significant during surgery. Therefore, a structural analysis module is to be developed to predict a scaffold’s mechanical properties such as overall stiffness and failure locations under stress. Lastly, capitalising on the semi-automatic nature of most procedures of the current workflow, a user-friendly minimal GUI is to be developed which will allow a surgeon to design a SGBR scaffold independently directly at the point of care.

## 5 Conclusion

The time efficient design of SGBR scaffolds is a critical requirement for routine clinical practice of this treatment method. The extended patient-specific scaffold design workflow demonstrated its application to three clinical case studies by successfully designing 3D printable patient-specific scaffold designs that can be inserted from the planned surgical approach without any obstruction from existing bone. The variability of the defect cases provide confidence in its applicability to a diverse range of clinical cases. The modular nature of the workflow further supports the time sensitive adoption to different clinical cases.

## Data Availability

The raw data supporting the conclusions of this article will be made available by the authors, without undue reservation. Requests to access the datasets should be directed to dietmar.hutmacher@qut.edu.au.

## Footnotes

1 3D printable: within the context of this study refers to a surface mesh being free from mesh errors that prevent 3D printing.

2 The QUT University Human Research Ethics Committee assessed this research as meeting the conditions for exemption from HREC review and approval in accordance with Section 5.1.17 of the National Statement on Ethical Conduct in Human Research (2023).

## References

[B1] Castrisos G. Gonzalez Matheus I. Sparks D. Lowe M. Ward N. Sehu M. (2022). Regenerative matching axial vascularisation of absorbable 3D-printed scaffold for large bone defects: a first in human series. J. plastic, Reconstr. and aesthetic Surg. JPRAS 75, 2108–2118. 10.1016/j.bjps.2022.02.057 35370116

[B2] Charbonnier B. Hadida M. Marchat D. (2021). Additive manufacturing pertaining to bone: hopes, reality and future challenges for clinical applications. Acta biomater. 121, 1–28. 10.1016/j.actbio.2020.11.039 33271354

[B3] Chen H. Han Q. Wang C. Liu Y. Chen B. Wang J. (2020). Porous scaffold design for additive manufacturing in orthopedics: a review. Front. Bioeng. Biotechnol. 8, 609. 10.3389/fbioe.2020.00609 32626698 PMC7311579

[B4] Chen H. Liu Y. Wang C. Zhang A. Chen B. Han Q. (2021). Design and properties of biomimetic irregular scaffolds for bone tissue engineering. Comput. Biol. Med. 130, 104241. 10.1016/j.compbiomed.2021.104241 33529844

[B5] Chen W. Dai N. Wang J. Liu H. Li D. Liu L. (2019). Personalized design of functional gradient bone tissue engineering scaffold. J. biomechanical Eng. 141, 111004. 10.1115/1.4043559 31017616

[B6] Cipitria A. Reichert J. C. Epari D. R. Saifzadeh S. Berner A. Schell H. (2013). Polycaprolactone scaffold and reduced rhBMP-7 dose for the regeneration of critical-sized defects in sheep tibiae. Biomaterials 34, 9960–9968. 10.1016/j.biomaterials.2013.09.011 24075478

[B7] Deering J. Dowling K. I. DiCecco L.-A. McLean G. D. Yu B. Grandfield K. (2021). Selective Voronoi tessellation as a method to design anisotropic and biomimetic implants. J. Mech. Behav. Biomed. Mater. 116, 104361. 10.1016/j.jmbbm.2021.104361 33550142

[B8] Du Y. Liang H. Xie D. Mao N. Zhao J. Tian Z. (2020). Design and statistical analysis of irregular porous scaffolds for orthopedic reconstruction based on voronoi tessellation and fabricated via selective laser melting (SLM). Mater. Chem. Phys. 239, 121968. 10.1016/j.matchemphys.2019.121968

[B9] Gatto M. L. Furlani M. Giuliani A. Bloise N. Fassina L. Visai L. (2021). Biomechanical performances of PCL/HA micro- and macro-porous lattice scaffolds fabricated via laser powder bed fusion for bone tissue engineering. Mater. Sci. and Eng. C, Mater. Biol. Appl. 128, 112300. 10.1016/j.msec.2021.112300 34474851

[B10] Ghadai S. Jignasu A. Krishnamurthy A. (2021). Direct 3D printing of multi-level voxel models. Addit. Manuf. 40, 101929. 10.1016/j.addma.2021.101929

[B11] Günther F. Wagner M. Pilz S. Gebert A. Zimmermann M. (2022). Design procedure for triply periodic minimal surface based biomimetic scaffolds. J. Mech. Behav. Biomed. Mater. 126, 104871. 10.1016/j.jmbbm.2021.104871 34654652

[B12] Henkel J. Medeiros Savi F. Berner A. Fountain S. Saifzadeh S. Steck R. (2021). Scaffold-guided bone regeneration in large volume tibial segmental defects. Bone 153, 116163. 10.1016/j.bone.2021.116163 34461285

[B13] Herath B. Laubach M. Suresh S. Schmutz B. Little J. P. Yarlagadda P. K. D. V. (2023). The development of a modular design workflow for 3D printable bioresorbable Patient-Specific bone scaffolds to facilitate clinical translation. Virtual Phys. Prototyp. 18. 10.1080/17452759.2023.2246434

[B14] Herath B. Suresh S. Downing D. Cometta S. Tino R. Castro N. J. (2021). Mechanical and geometrical study of 3D printed voronoi scaffold design for large bone defects. Mater. and Des. 212, 110224. 10.1016/j.matdes.2021.110224

[B15] Jia Z. Xu X. Zhu D. Zheng Y. (2023). Design, printing, and engineering of regenerative biomaterials for personalized bone healthcare. Prog. Mater. Sci. 134, 101072. 10.1016/j.pmatsci.2023.101072

[B16] Kambampati S. Jauregui C. Museth K. Kim H. A. (2021). Geometry design using function representation on a sparse hierarchical data structure. Computer-aided Des. Appl. 133, 102989. 10.1016/j.cad.2020.102989

[B17] Karakoç A. (2021). RegionTPMS — region based triply periodic minimal surfaces (TPMS) for 3-D printed multiphase bone scaffolds with exact porosity values. SoftwareX 16, 100835. 10.1016/j.softx.2021.100835

[B18] Kikinis R. Pieper S. D. Vosburgh K. G. (2014). “3D slicer: a platform for Subject-Specific image analysis, visualization, and clinical support,” in Intraoperative imaging and image-guided therapy. Editor Jolesz F. A. (New York, NY: Springer New York), 277–289. 10.1007/978-1-4614-7657-3_19

[B19] Klatt M. A. Lovrić J. Chen D. Kapfer S. C. Schaller F. M. Schönhöfer P. W. A. (2019). Universal hidden order in amorphous cellular geometries. Nat. Commun. 10, 811. 10.1038/s41467-019-08360-5 30778054 PMC6379405

[B20] Kobbe P. Laubach M. Hutmacher D. W. Alabdulrahman H. Sellei R. M. Hildebrand F. (2020). Convergence of scaffold-guided bone regeneration and RIA bone grafting for the treatment of a critical-sized bone defect of the femoral shaft. Eur. J. Med. Res. 25, 70. 10.1186/s40001-020-00471-w 33349266 PMC7754593

[B21] Laubach M. Hildebrand F. Suresh S. Wagels M. Kobbe P. Gilbert F. (2023). The concept of scaffold-guided bone regeneration for the treatment of long bone defects: current clinical application and future perspective. J. Funct. biomaterials 14, 341. 10.3390/jfb14070341 PMC10381286 37504836

[B22] Laubach M. Suresh S. Herath B. Wille M.-L. Delbrück H. Alabdulrahman H. (2022). Clinical translation of a patient-specific scaffold-guided bone regeneration concept in four cases with large long bone defects. J. Orthop. Transl. 34, 73–84. 10.1016/j.jot.2022.04.004 PMC9213234 35782964

[B23] Minetto R. Volpato N. Stolfi J. Gregori R. M. M. H. da Silva M. V. G. (2017). An optimal algorithm for 3D triangle mesh slicing. Computer-aided Des. Appl. 92, 1–10. 10.1016/j.cad.2017.07.001

[B24] Museth K. (2013). VDB: high-resolution sparse volumes with dynamic topology. ACM Trans. Graph. 32, 1–22. 10.1145/2487228.2487235

[B35] National Statement on Ethical Conduct in Human Research (2023). consists of a series of guidelines made in accordance with the National Health and Medical Research Council Act 1992.

[B25] Onal E. Frith J. E. Jurg M. Wu X. Molotnikov A. (2018). Mechanical properties and *in vitro* behavior of additively manufactured and functionally graded Ti6Al4V porous scaffolds. Metals 8, 200. 10.3390/met8040200

[B26] Poltue T. Karuna C. Khrueaduangkham S. Seehanam S. Promoppatum P. (2021). Design exploration of 3d-printed triply periodic minimal surface scaffolds for bone implants. Int. J. Mech. Sci. 211, 106762. 10.1016/j.ijmecsci.2021.106762

[B27] Popescu D. Marinescu R. Laptoiu D. Deac G. C. Cotet C. E. (2021). DICOM 3D viewers, virtual reality or 3D printing – a pilot usability study for assessing the preference of orthopedic surgeons. Proc. Institution Mech. Eng. Part H, J. Eng. Med. 235, 1014–1024. 10.1177/09544119211020148 34176364

[B28] Reichert J. C. Cipitria A. Epari D. R. Saifzadeh S. Krishnakanth P. Berner A. (2012). A tissue engineering solution for segmental defect regeneration in load-bearing long bones. Sci. Transl. Med. 4, 141ra93. 10.1126/scitranslmed.3003720 22764209

[B29] Sharma N. Ostas D. Rotar H. Brantner P. Thieringer F. M. (2021). Design and additive manufacturing of a biomimetic customized cranial implant based on voronoi diagram. Front. physiology 12, 647923. 10.3389/fphys.2021.647923 PMC8063040 33897455

[B30] Shi J. Zhu L. Li L. Li Z. Yang J. Wang X. (2018). A TPMS-based method for modeling porous scaffolds for bionic bone tissue engineering. Sci. Rep. 8, 7395. 10.1038/s41598-018-25750-9 29743648 PMC5943328

[B31] Verma R. Kumar J. Singh N. K. Rai S. K. Saxena K. K. Xu J. (2022). Design and analysis of biomedical scaffolds using TPMS-based porous structures inspired from additive manufacturing. Coatings World 12, 839. 10.3390/coatings12060839

[B32] Wesorick B. Kelly C. Gall K. (2024). “2 - CT to software and other considerations,” in Clinical applications of 3D printing in foot and ankle surgery First Edition (New Delhi: Elsevier), 17–24. 10.1016/B978-0-323-82565-8.00011-1

[B33] Yang Y. P. Labus K. M. Gadomski B. C. Bruyas A. Easley J. Nelson B. (2021). Osteoinductive 3D printed scaffold healed 5 cm segmental bone defects in the ovine metatarsus. Sci. Rep. 11, 6704. 10.1038/s41598-021-86210-5 33758338 PMC7987996

[B34] Zhou J. See C. W. Sreenivasamurthy S. Zhu D. (2023). Customized additive manufacturing in bone scaffolds—the gateway to precise bone defect treatment. Research/ a J. Sci. its Appl. 6, 0239. 10.34133/research.0239 PMC10561823 37818034

