# Supplementary material for: Modular design workflow for 3D printable bioresorbable patient-specific bone scaffolds: extended features and clinical validation
Source: Front Bioeng Biotechnol. 2024 Nov 19;12:1404481. doi: 10.3389/fbioe.2024.1404481 (PMC11611564; doi:10.3389/fbioe.2024.1404481)
Supplement: Supplementary file 1 [file DataSheet1.pdf]

frontiers

Supplementary Material

1 DEMONSTRATION VIDEO OF THE SURGICAL RESECTION MODULE

Demonstration video of the Surgical Resection module:
https://www.frontiersin.org/journals/bioengineering-and-biotechnology/article[illegible]
10.3389/fbioe.2024.1404481/abstract#supplementary-material. The playback speed has not been increased and is in real-time.

2 SURGICAL STRATEGIES OF CASE 1 AND 2

Surgical strategies of the on-going clinical cases whose scaffolds were surgically implanted are discussed in detail here.

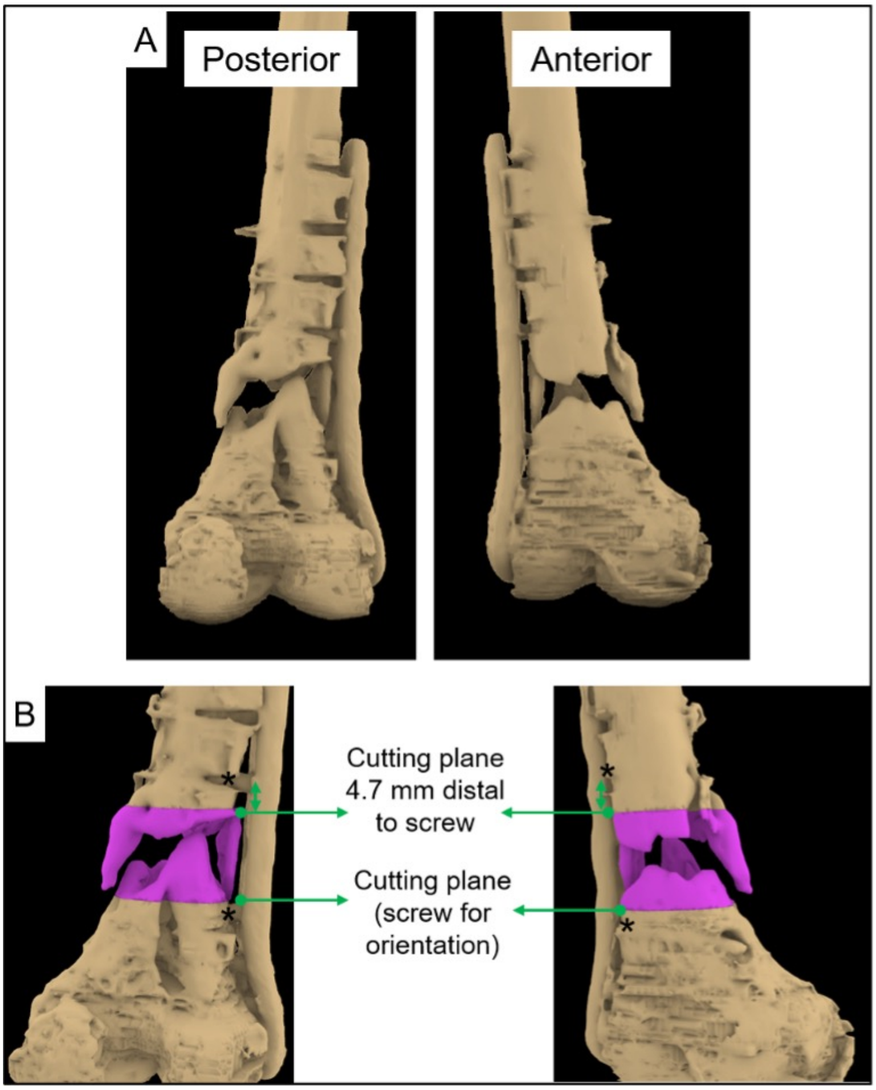

Figure S1. Case 1 right femur: 3D reconstruction of the pre-operative osseous status (A) with corresponding planning of resection of sclerotic and partially necrotic bone parts (B). Please note, the inserted osteosynthesis material was considered stable so that the inserted screws also served as anatomical landmarks during surgery. Asterisks (*) in (B) indicate screws.

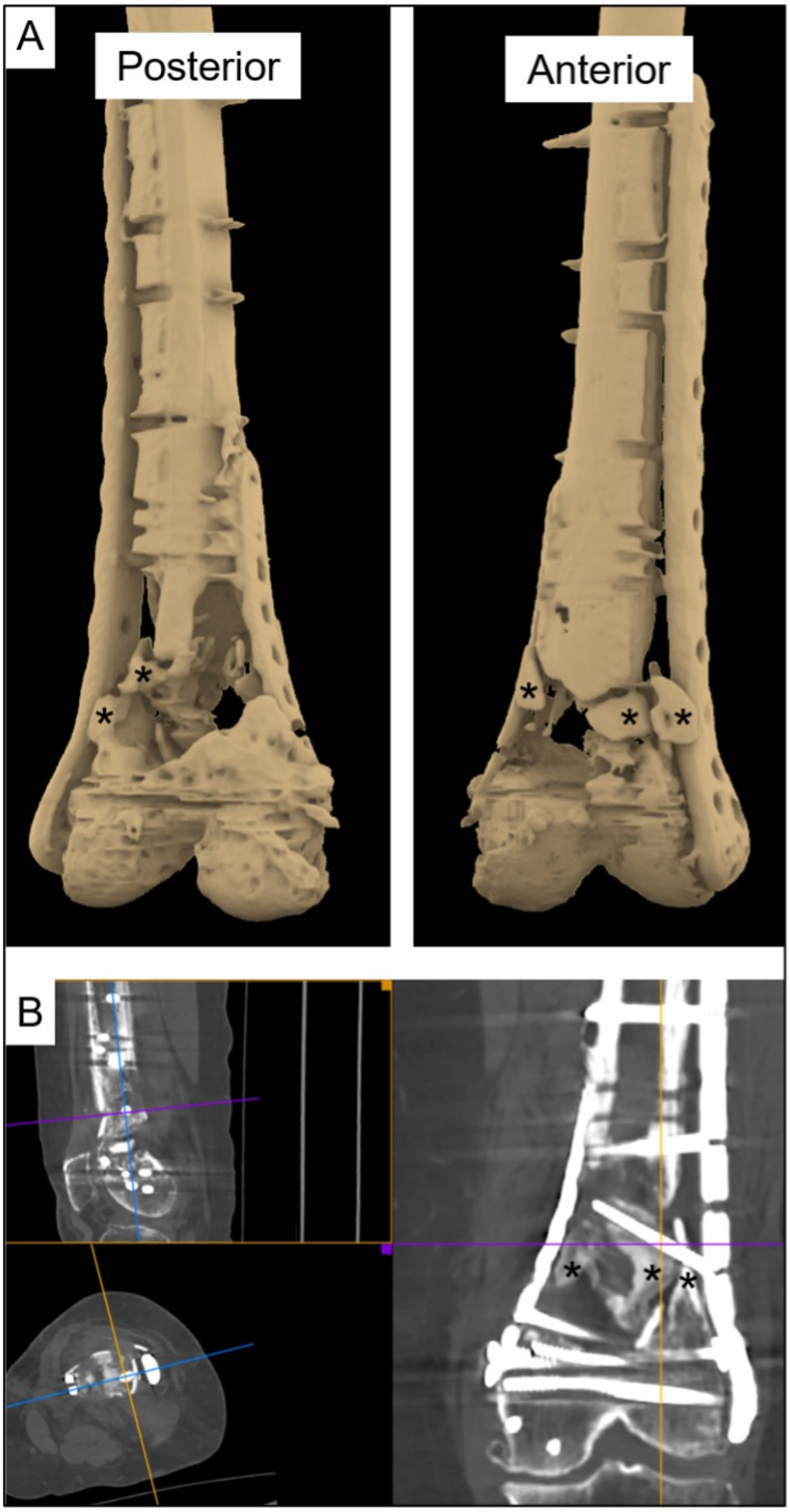

Figure S2. Case 1 left femur (1/3): 3D reconstruction of CT data in the area of the bone defect in the area of the distal left femur showed sclerosed and necrotic bone fragments (A). Furthermore, the CT analysis showed several screws in the area of the bone defect, which therefore no longer contributed to the biomechanical stabilization (B). Asterisks (*) indicate sclerotic/necrotic bone.

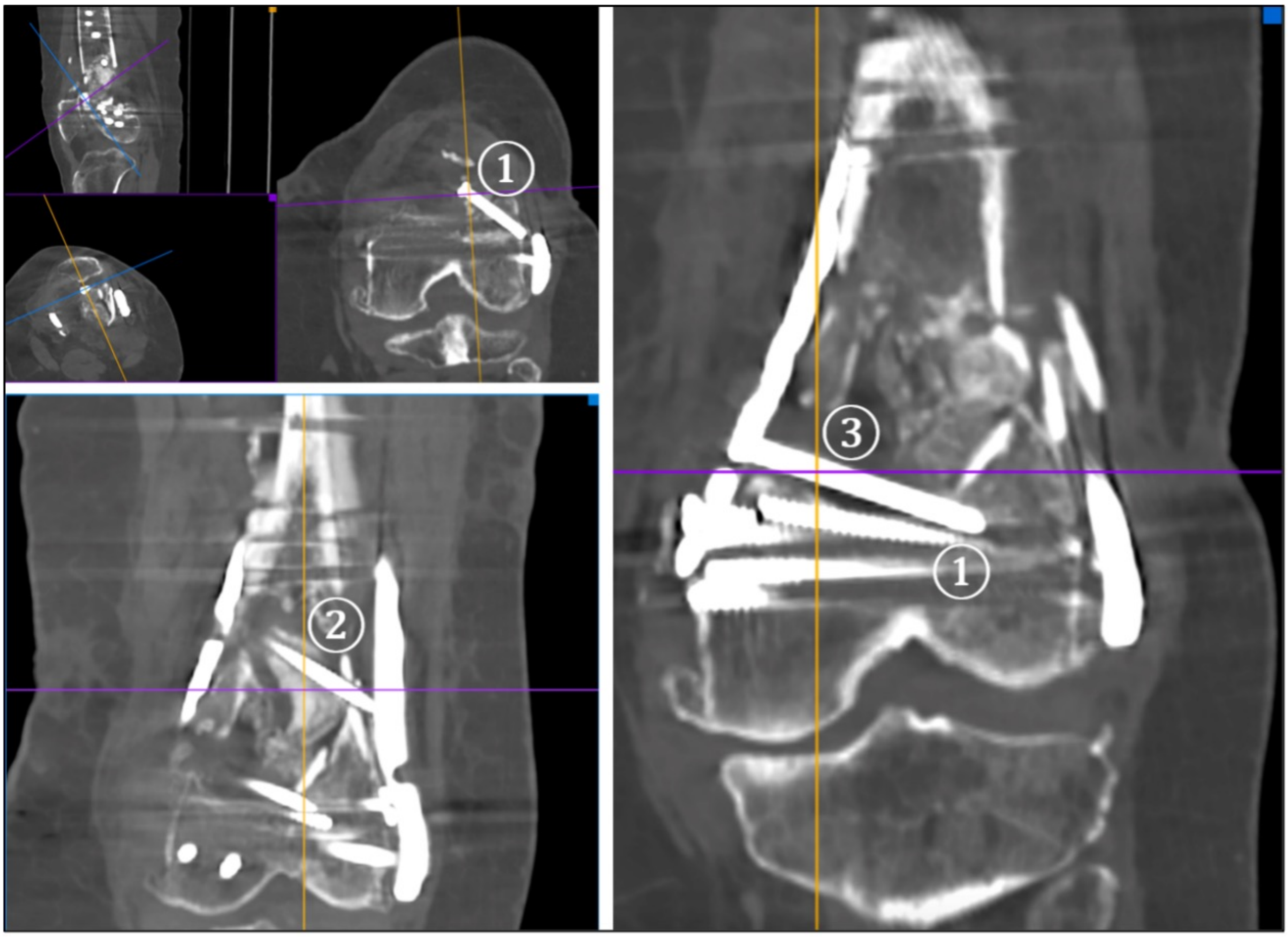

Figure S3. Case 1 left femur (2/3): It was necessary to remove 3 inserted screws or screw fragments in the course of resection of sclerosed and necrotic bone parts. The respective screws are indicated with the numbers 1-3, where (1) was a broken screw and (2) and (3) were inserted from the medial and lateral directions, respectively.

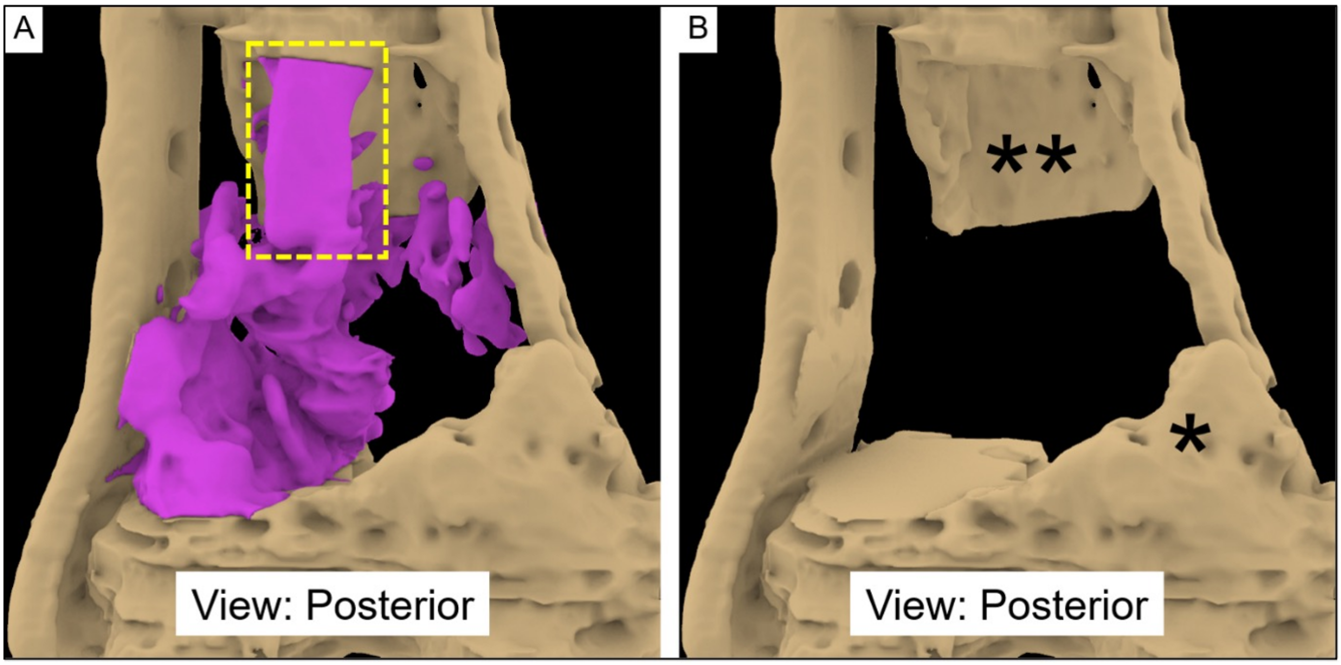

Figure S4. Case 1 left femur (3/3): The bone fragments marked in purple colour are considered sclerotic or necrotic due to their morphology and partial dislocation from the host bone (A). In particular, it was decided to remove a posteriorly located protruding bone fragment as early as the planning phase (dashed rectangle in A). In the planning phase, after the planned removal of the bone fragments as shown in A, the residual bone (* in B) showed that it was very likely to have good vitality and that there was sufficient space to insert modular scaffolds (B). Please note it was planned to keep a larger protruding bone fragment anteriorly (** in B) which required designing of modular scaffolds.

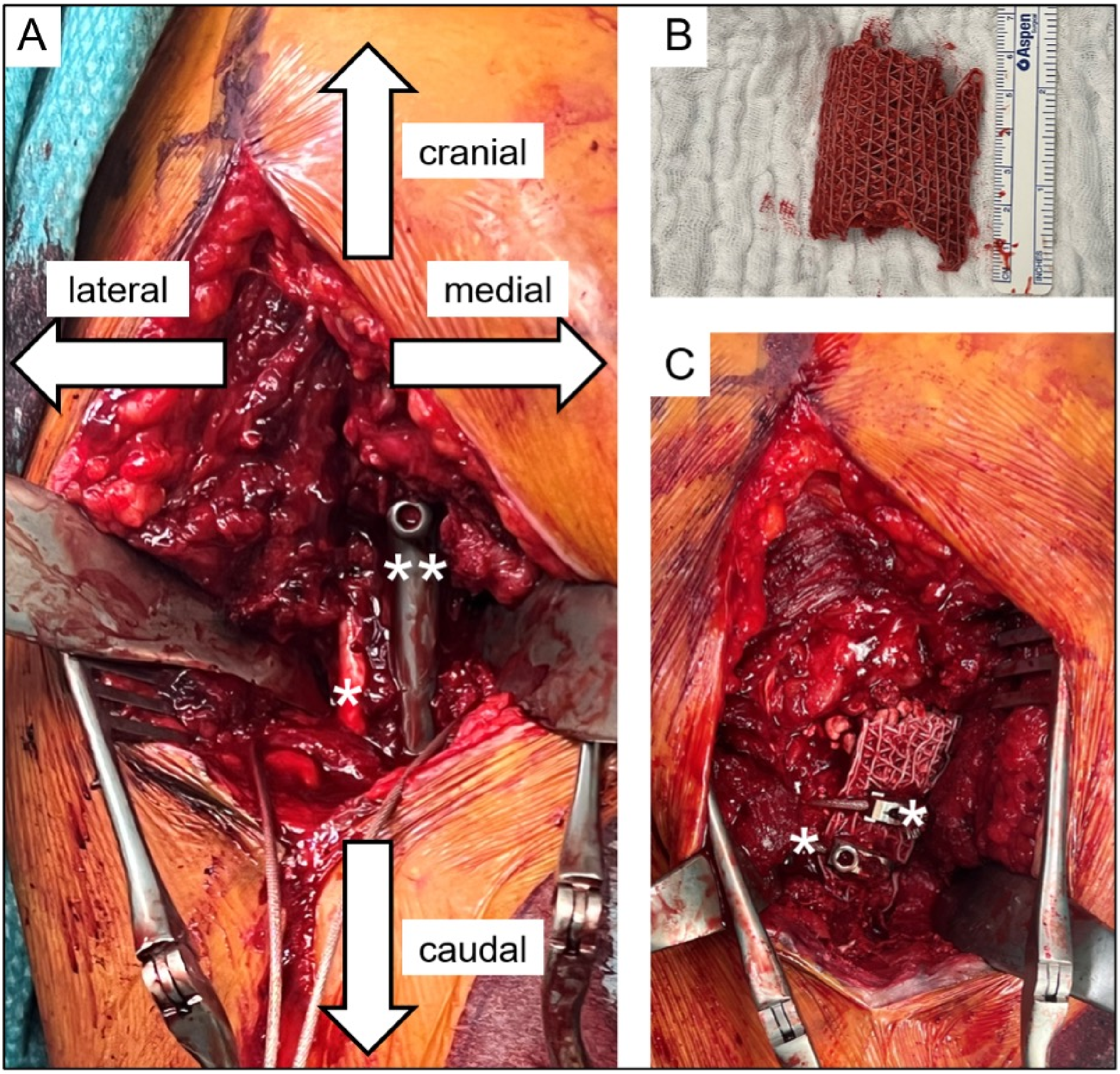

Figure S5. Case 2 left humerus: After routine antero-lateral approach to the proximal humerus, the preceding radiological imaging was confirmed and only partial bony coverage (*) of an exposed intramedullary nail (**), especially in the anterior region, was observed (A). Due to highly exposed neurovascular structures in the medial region of the situs, it was decided intraoperatively to implant only 1 scaffold, in contrast to the preoperative planning. For this purpose, the standard preparation of the scaffold and its loading with autologous bone graft (B) was performed. The scaffold fitted tightly and was additionally stabilized with cerclages (*) (C).

3 SCAFFOLDS MANUFACTURED BY THE CERTIFIED MANUFACTURER

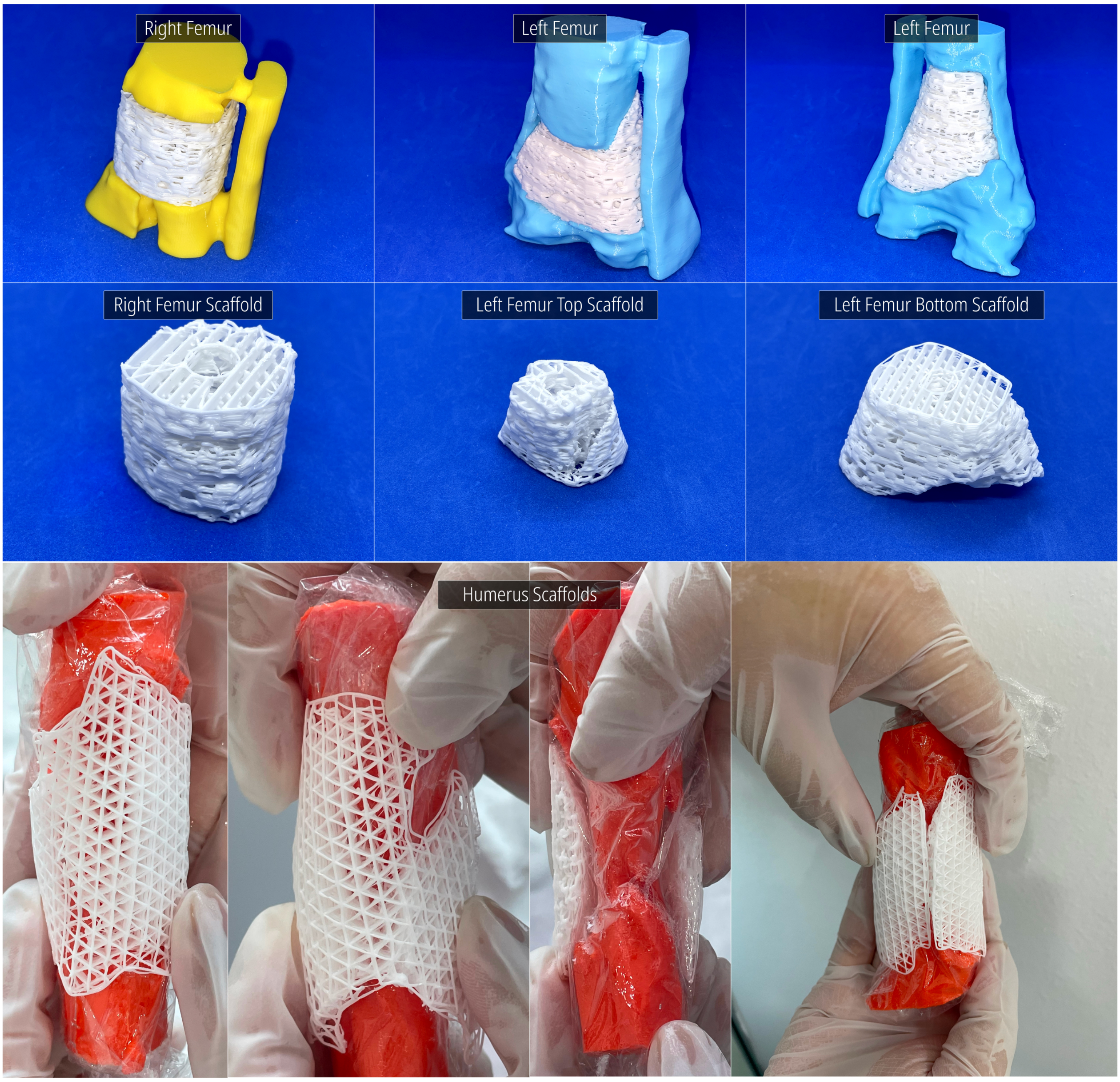

Figure S6. The scaffolds 3D printed under sterile conditions by the certified manufacturer using the rectilinear infill (alternating layers of 0°, 60°, 120° degrees) architecture. The manufacturer printed the scaffolds slightly thicker than what was designed as a standard practice to cater for material shrinkage (naturally occurring) and to adjust for any potential preoperative bone loss due to bone-resorption. Hence, the surgeons were advised that they may or may not need to trim the scaffolds to ensure a proper fit.

4 ALTERNATIVE APPROACH TO DICOM OVERLAY REGISTRATION MODULE

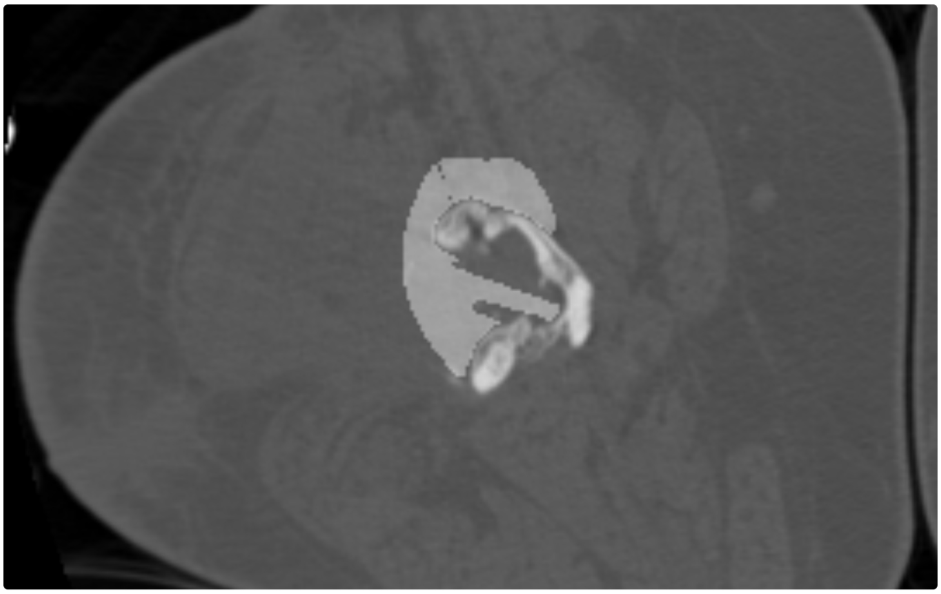

Figure S7. The greyscale version of the DICOM Overlay Registration module. This version allows the embedding of the scaffold designs in the DICOM stack itself (as opposed to a different SEG object) which allows the surgeon to view the stack using any DICOM viewer they prefer. However, for complicated cases, clarity could be compromised.
